# The transcription factor GATA10 regulates fertility conversion of a two‐line hybrid *tms5* mutant rice *via* the modulation of *Ub*_*L40*_ expression

**DOI:** 10.1111/jipb.12871

**Published:** 2019-11-06

**Authors:** Jing Jin, Songtao Gui, Qian Li, Ying Wang, Hongyuan Zhang, Zhixuan Zhu, Hao Chen, Yueyang Sun, Yu Zou, Xingguo Huang, Yi Ding

**Affiliations:** ^1^ State Key Laboratory of Hybrid Rice, Department of Genetics, College of Life Sciences Wuhan University Wuhan 430072 China; ^2^ Institute of Vegetable Wuhan Academy of Agricultural Sciences Wuhan 430072 China; ^3^ Wuhan Wuda Tianyuau Bio‐Tech Co., Ltd. Wuhan 430070 China

## Abstract

The *thermosensitive genic male sterile 5* (*tms5*) mutation causes thermosensitive genic male sterility in rice (*Oryza sativa*) through loss of RNase Z^S1^ function, which influences *ubiquitin fusion ribosomal protein L40* (*Ub*_*L40*_) messenger RNA levels during male development. Here, we used ATAC‐seq, combined with analysis of H3K9ac and H3K4me2, to identify changes in accessible chromatin during fertility conversion of the two‐line hybrid rice Wuxiang S (WXS) derived from a mutant *tms5* allele. Furthermore, RNA‐seq and bioinformatic analyses identified specific transcription factors (TFs) in differentially accessible chromatin regions. Among these TFs, only GATA10 targeted *Ub*_*L40*_. *Osgata10* knockout mutations, which resulted in low expression of *Ub*_*L40*_ and a tendency toward male fertility, confirmed that GATA10 regulated fertility conversion *via* the modulation of *Ub*_*L40*_. Meanwhile, GATA10 acted as a mediator for interactions with ERF65, which revealed that transcriptional regulation is a complex process involving multiple complexes of TFs, namely TF modules. It appears that the ERF141/MADS7/MADS50/MYB modules affect metabolic processes that control anther and pollen development, especially cell wall formation. Our analysis revealed that these modules directly or indirectly affect metabolic pathway‐related genes to coordinate plant growth with proper anther development, and furthermore, that GATA10 regulates fertility conversion *via* the modulation of *Ub*_*L40*_ expression.

## Graphical abstract

Accessible chromatin profiling of a two‐line hybrid *tms5* mutant rice was identified to understand transcriptional regulation and uncover key regulatory modules. Our study revealed that these modules affect metabolic pathway‐related genes to control anther development, and furthermore, that GATA10 regulates fertility conversion via the modulation of *Ub_L40_* expression.

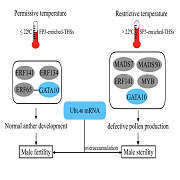

## INTRODUCTION

Hybrid rice, which has contributed to improving rice yields, is usually produced using either a three‐line or two‐line system (Cheng et al. 2007). A three‐line system uses a cytoplasmic male sterility line, a maintainer line, and a restorer line, whereas a two‐line system uses a photoperiod‐sensitive genic male‐sterile (PGMS) or thermosensitive genic male‐sterile (TGMS) line. Two‐line systems are superior to three‐line systems owing to the lack of requirement for maintainer lines, lack of cytoplasmic effects, lower costs, and higher yields (Deng et al. 1999; Wang et al. 2006). Photoperiod‐sensitive genic male‐sterile and thermosensitive genic male‐sterile lines are male‐sterile under restrictive conditions (long‐day for PGMS and high temperatures for TGMS) but convert to male‐fertile lines under permissive conditions (short‐day for PGMS and low temperatures for TGMS) (Zhou et al. 2012). Therefore, the male‐sterile line of the two‐line system frequently suffers from unpredictable conversion from sterility to fertility due to environmental fluctuation, which has a major impact on hybrid seed production. Enhanced molecular understanding is critical for maintaining the stability of the system in a fluctuating environment, thus further improving two‐line hybrid breeding of rice and other crops (Fan and Zhang 2017).

TGMS lines are more widely used than PGMS lines in two‐line hybrid rice breeding, and *thermosensitive genic male sterile 5* (*tms5*) serves as the major TGMS gene (Si et al. 2012). *TMS5* encodes a conserved RNase Z^S1^ protein that can process the three messenger RNAs (RNAs) of genes of the ubiquitin‐60S ribosomal protein L40 family (*Ub*_*L40*_), namely, *Ub*_*L40*_ *1, Ub*_*L40*_ *2, and Ub*_*L40*_ *4*, into multiple fragments. In *tms5* mutants, the overaccumulation of *Ub*_*L40*_ mRNAs caused by high temperature leads to defective pollen production and male sterility (Zhou et al. 2014). In addition, the transcription factor (TF) OsbHLH138 can regulate the expression of *tms5* and the accumulation of *Ub*_*L40*_ mRNAs to control male fertility in different temperatures, indicating some TFs play vital roles during fertility conversion (Wen et al. 2019). To date, a variety of other PGMS/TGMS genes with various origins have been discovered, including *tms6*, *tms9*, *pms3, ptms12* and others (Wang et al. 2003; Zhou et al. 2012), but relatively little is known about the transcriptional regulatory networks that may coordinate the timing and rate of genome‐wide gene expression in response to environmental and developmental signals.

In eukaryotic nuclei, DNA wraps around core histones to form nucleosomes, which are organized into higher‐order structures to form chromatin (Li et al. 2007). The chromatin structure plays a pivotal role in genome organization and transcriptional regulation. Nucleosome‐depleted chromatin regions are regarded as *cis*‐regulatory DNA elements, such as promoters and enhancers, that are usually targeted by TFs (Berger 2007). Some TFs can bind to their *cis*‐regulatory regions in nucleosomal DNA by recruiting chromatin remodelers that open up nucleosomes; this action facilitates the binding of regulatory proteins and their interactions in regulatory networks, which mainly control the activation or repression of the expression of nearby target genes (Zaret and Carroll 2011). Plants can respond acutely to environmental fluctuations, resulting in differentially expressed genes (DEGs) established by different regulatory landscapes at the transcriptional and epigenetic levels; however, the *cis*‐regulatory elements and TFs of regulatory networks controlling these changes remain unclear. Accurate knowledge about the regulatory motifs bound by TFs in their native chromatin state in a particular condition can provide vital insight into the mechanisms of transcriptional regulation. Numerous studies have shown that TF binding is correlated with epigenetic phenomena such as histone modification and DNase I accessibility (Zentner and Henikoff 2013). For instance, histone modifications have been identified as regulators of gene expression through changes in the chromatin state and recruitment of regulated proteins (Schneider et al. 2004; He et al. 2010). Chromatin accessibility was used together with the knowledge of *cis*‐regulatory motifs to identify a regulatory network of the response of *Arabidopsis thaliana* to high temperature and during photomorphogenesis (Sullivan et al. 2014; Zhang et al. 2015). The assay for transposase‐accessible chromatin using sequencing (ATAC‐seq) is an emerging method (Buenrostro et al. 2013, 2015). The advantages of this method make it superior to traditional DNaseI‐seq methods, such as its simple two‐step protocols, high sensitivity with small amounts of starting materials and ability to simultaneously examine multiple aspects of chromatin architecture. The relative simplicity of the ATAC‐seq procedure and the low quantity of nuclei required, combined with its recent application in *Arabidopsis* and rice, has made ATAC‐seq widely useful for examining plant DNA regulatory regions (Maher et al. 2018; Sijacic et al. 2018).

In the present study, we used a novel photo‐thermo‐sensitive genic male‐sterile (PTGMS) line, Wuxiang S (WXS), which was generated by our laboratory and derived from an *indica* rice line with a mutant *tms5* locus (Zhou et al. 2014; Zhang et al. 2016). At the restrictive temperature (>22°C), we collected young panicles of male‐sterile WXS (designated WXS(S)) at the meiosis period (P3) and the uninucleate period (P4). Meanwhile, at the permissive temperature (≤22°C), we collected young panicles of male‐fertile WXS (WXS(F)) at P3 and P4. Then, we used ATAC‐seq along with assays for H3K9ac and H3K4me2 to identify the changes in chromatin accessibility between four distinct panicle tissues of WXS (i.e., WXS(S) and WXS(F) each at P3 and P4). Further, the differential *cis*‐regulatory elements occupied by specific TFs were combined with our RNA sequencing (RNA‐seq) data derived from the same tissues used for ATAC‐seq. These specific TFs, which include ERF141, MADS7, MADS50, and MYB, appear to play a vital role in fertility conversion by regulation of downstream target genes to control anther and pollen development. Among these specific TFs, transgenic plants containing a knockout of *Osgata10* produced pollen without starch deposition and exhibited significantly reduced levels of *Ub*_*L40*_ *1* and *Ub*_*L40*_ *4* mRNA, indicating that GATA10 directly regulated fertility conversion by modulating downstream *Ub*_*L40*_ mRNA expression, which partly explained why *Ub*_*L40*_ mRNA could overaccumulate at high temperatures. In addition, GATA10 acted as a mediator of interaction with WXS(F)‐P3‐enriched TF ERF65, which reveals that transcriptional regulation is a complex process involving multiple complexes of TFs, namely TF modules that affect many biological processes during fertility conversion. Our data highlight that ATAC‐seq combined with gene expression analysis is a powerful technique: in the present study, it revealed that GATA10 regulates fertility conversion of two‐line hybrid *tms5* mutant rice *via* the modulation of *Ub*_*L40*_ expression and complements the transcriptional regulatory mechanism of *Ub*_*L40*_ expression.

## RESULTS

### The meiosis and the uninucleate periods serve as key periods of fertility conversion

The young panicles of WXS(F) and WXS(S) were collected at different developmental stages based on panicle length and cross section (Zhang and Wilson 2009). To determine the critical periods of WXS fertility conversion, we performed cytological observations. WXS(F) had normal anthers and mature pollen that stained deeply with iodine‐potassium iodide (I_2_‐KI) (1A, C); however, WXS(S) with abnormal anthers showed the non‐pollen type of sterility (1B, D). Subsequently, anther transverse sections of WXS(F) showed that the microspore mother cells (MMCs) completed meiosis to form normal tetrads of haploid microspores, and the tapetal cells began to degenerate, ultimately producing mature pollen (1E–K). By contrast, the MMCs of WXS(S) underwent abnormal meiosis and cell division, resulting in aberrant tetrads and microspores, while the tapetal cells became abnormally enlarged. The pollen eventually disintegrated into debris, and the tapetal cells persisted (1L–R). We chose two critical panicle development periods with obvious cytological differences between WXS(F) and WXS(S), the meiosis period (P3; 1F, M) and the uninucleate period (P4; 1I–Q), to perform ATAC‐seq, to obtain genome‐wide accessible chromatin profiling and to further uncover new transcription regulatory modules involved in fertility conversion.

**Figure 1:**
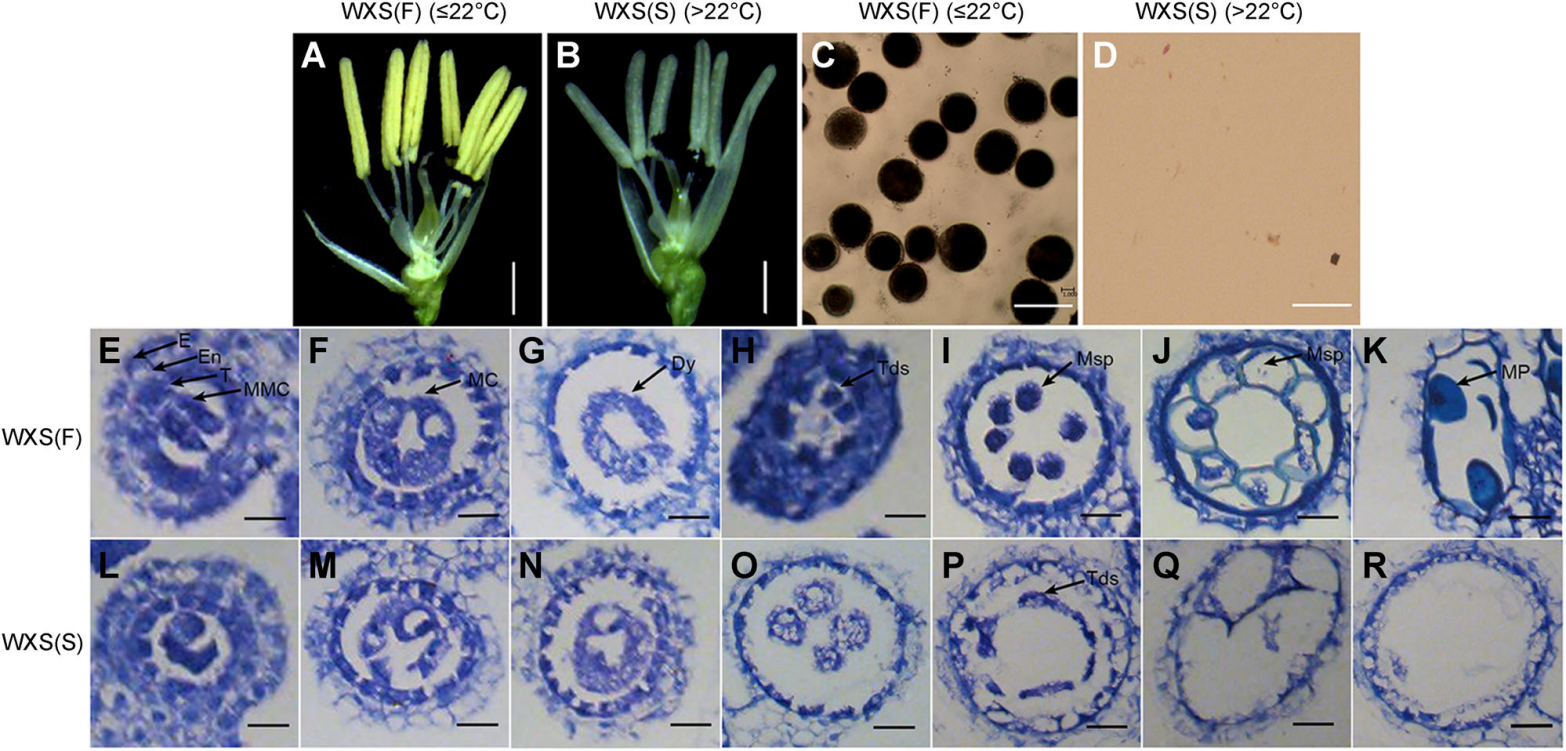
**Phenotypic and cytological observation of WXS(F) and WXS(S)** **(A**–**D**) Anther morphology and pollen fertility of WXS(F) and WXS(S). (**A, C**) WXS(F) with normal anthers and fertile pollen at the permissive temperature (≤22°C). (**B, D**) WXS(S) with abnormal anthers and no pollen at the restrictive temperature (>22°C). (**E–R**) Comparison of transverse sections of the anthers of WXS(F) and WXS(S). (**E, L**) Microspore mother cell formation stage; (**F, M**) meiosis stage; (**G, N, O**) dyad stage; (**H, P**) tetrad stage; (**I, J, Q**) young microspore stage; (**K, R**) mature pollen stage. E, epidermis; En, endothecium; T, tapetum; MMC, microspore mother cell; MC, meiotic cell; Dy, dyad cell; Tds, tetrads; Msp, microspore parietal cell; MP, mature pollen. Scale bar: (**A, B**) 1 mm, (**C, D**) 5 µm and (**E–R**) 30 µm.

### Accessible chromatin profiling in rice panicles by ATAC‐seq with additional epigenetic markers

Accessible chromatin regions of the genome are *cis*‐regulatory elements occupied by TFs that regulate gene expression in nearby DNA regions (Natarajan et al. 2012). We performed ATAC‐seq to identify accessible chromatin regions of the young panicles of WXS(F) and WXS(S) at P3 and P4, and the isolated nuclei were confirmed to be sufficient and intact (Figure S1A). Our results showed that approximately 25% of all reads were successfully mapped to the nuclear genome, and the remainder (which mapped to organelle genomes) were omitted from downstream analyses. More than 42 million reads per replicate passed the quality filtering stage of analysis (Figure S1B), which was sufficient to successfully identify accessible chromatin regions in rice, as has been recently illustrated (Wilkins et al. 2016). The fragment size distribution of the aligned reads was used to distinguish the number of nucleosome‐containing (>150 bp) and nucleosome‐free reads (<150 bp) (Sijacic et al. 2018). In our ATAC‐seq datasets, the fragment size distribution showed a significant peak at 50‐bp fragments with a clear pattern of the helical pitch of DNA, indicating that our libraries were composed predominately of nucleosome‐free reads and were of sufficient coverage (Figure S1C).

In addition, the read intensities of ATAC‐seq were highly reproducible between the two biological replicates (*R* ^2^ = 0.98) (2A), which indicated that small amounts of complex, multi‐tissue rice panicles (approximately 0.5 g) were sufficient to obtain high‐quality accessible chromatin profiling. Our ATAC‐seq data for all four rice samples overlapped well with published TIGR7 reference genome DNaseI hypersensitive sites (TIGR7_DHS) obtained from the Plant DHS database (http://plantdhs.org) (Figure S1D). Collectively, these data suggest that ATAC‐seq provides a highly sensitive method to examine accessible chromatin regions and *cis*‐regulatory element activities in the panicles of rice WXS.

**Figure 2:**
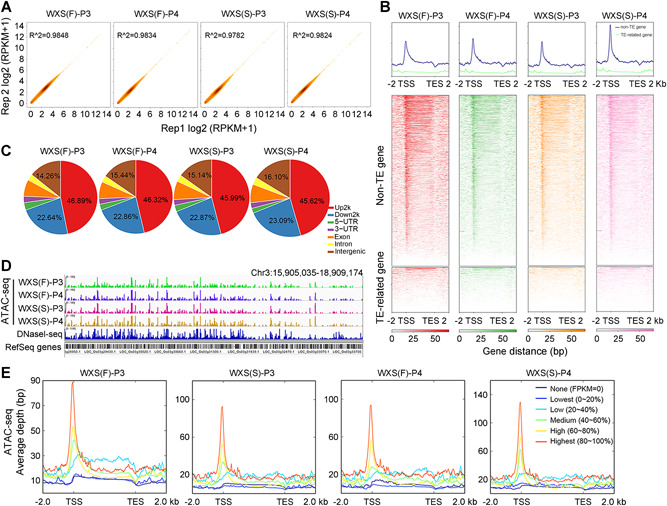
**Accessible chromatin profiling by ATAC‐seq and comparison with RNA‐seq data** **(A)** Scatter plots comparing the enrichment between two ATAC‐seq replicates. Pearson correlation of the reads per kilobase per million mapped reads (RPKM) values is shown. **(B)** Average plots and heat maps of ATAC‐seq signals in transposable element (TE)‐related and non‐TE‐related genes. **(C)** Distribution of all Tn5 transposase hypersensitive sites (THSs) in the rice genome. (up2k) 2‐kb region upstream of transcription start site (TSS). (down2k) 2‐kb region downstream from the end of gene transcription (TES). **(D)** Visualization by Integrative Genomics Viewer (IGV) showing THS enrichment of ATAC‐seq in all four WXS samples, as well as DNaseI hypersensitive site enrichment in the former published DNase‐seq data in rice. Gene models are displayed on the bottom track. **(E)** The profile of Tn5 transposase sensitivity (indicated by the number of ATAC‐seq reads) among the genes with different expression levels from our RNA‐seq data. The expressed genes were divided into five bins from lowest to highest expression.

To refer to accessible chromatin regions by model‐based analysis for ChIP‐seq (MACS) as Tn5 transposase hypersensitive sites (THSs), that is, peak numbers, herein we identified about 59,900 THSs covering approximately 40 Mb (approximately 10% of the genome) (Figure S1E). The genomic distribution of the THSs showed a very similar pattern among the four samples (WXS(F) and WXS(S) at P3 and P4), with the 2‐kb region upstream of TSS (up2k region) occupying a major part, followed by the 2‐kb region downstream from the end of gene transcription (down2k) and intergenic regions (2C), in agreement with previous findings showing that promoters were associated with accessible chromatin in plant genomes (Du et al. 2013). Furthermore, we investigated the changes in amount of accessible chromatin and observed that only the accessible chromatin regions of non‐transposable‐element (TE) genes were enriched, mostly upstream of the transcription start sites (TSS) (2B); this is consistent with the majority of the *cis*‐regulatory elements in the rice genome being located near gene core promoters, as previously observed in other plants (Maher et al. 2018). Visualization of these THSs in a genome browser, along with the TIGR7_DHS data obtained for rice DNaseI‐seq data, revealed a high overall degree of similarity among the five datasets in this study (i.e., ATAC‐seq data for the four samples plus the published DNaseI‐seq data; 2D). When compared with our RNA‐seq data sets derived from the same tissues used for ATAC‐seq, the expression levels of the rice genes were positively correlated with the levels of ATAC‐seq signals upstream of the TSS (2E). Genes with higher levels of expression displayed higher levels of Tn5 transposase sensitivity within this region.

A compelling question concerns the cause of the chromatin regions to be open or closed during fertility conversion. The epigenetic modification patterns of the histone mark distribution of these genomic regions may provide some clues for answering this question. Chromatin immunoprecipitation sequencing (ChIP‐seq) was used to assay for H3K4me2 and H3K9ac (Li et al. 2008; Du et al. 2013), and then those data were integrated with our ATAC‐seq and RNA‐seq data to further profile the global landscape for diverse regulatory elements and gain an integrated map of the epigenome and transcripts during fertility conversion (Figure S2A). Based on the above results, we determined that the read intensities of ChIP‐seq and ATAC‐seq showed relatively high replicate consistency (*R* ^2^ ≥ 0.5) (Figure S2B). Both H3K9ac and H3K4me2 were predominantly enriched around the TSSs, which showed a similar pattern to the ATAC‐seq enrichment; however, H3K4me2 was also enriched in the gene body (Figure S2C). In addition, the relationship between transcript levels and epigenetic modifications indicated a linear positive relationship between H3K9ac levels and transcript abundance, whereas no correlation was detected between H3K4me2 and transcript abundance (Figure S2C). To confirm the reliability of ChIP‐seq, we randomly chose 14 genes with or without differential H3K9ac and H3K4me2 enrichment between WXS(F) and WXS(S) to perform quantitative real‐time polymerase chain reaction (qRT‐PCR), all of which showed the same enrichment patterns observed in the ChIP‐seq data (Figure S3). These results suggest that H3K4me2 correlates with the permissive state of chromatin, in which genes are either active or potentially active. In addition, potentially active genes with the H3K4me2 mark under the permissive state of chromatin can easily be regulated by TFs.

### Commonalities and distinctions in accessible chromatin landscapes

Pairwise comparisons of WXS(S)‐P3 (SP3), WXS(S)‐P4 (SP4), WXS(F)‐P3 (FP3), and WXS(F)‐P4 (FP4) by MAnorm were used to identify regions of differential accessibility between different periods (P3 vs. P4) and different sample types (S vs. F). Only those THSs that had a |M‐value| greater than 1 were categorized as THSs enriched in that sample type, which were referred to as differential THSs (dTHSs) (Table S1). To identify genes that might be controlled by the dTHSs, we mapped each dTHS to its nearest TSS and considered that to be the target gene (Table S2). In the comparison between SP3 and FP3, we identified 11,849 SP3‐enriched dTHSs that mapped to 5,467 genes, while there were 1,980 FP3‐enriched dTHSs that mapped to 2,261 genes. Moreover, in the comparison between SP4 and FP4, we observed 3,519 SP4‐enriched dTHSs that mapped to 2,090 genes, while there were 1,375 FP4‐enriched dTHSs that mapped to 1,081 genes (Table S2).

We further examined whether these dTHS‐associated genes had a relationship with the DEGs from our RNA‐seq data sets. In FP3, 2,842 and 1,091 genes were respectively up‐ and downregulated more than 2‐fold or greater difference in abundance (*P*‐value < 0.05) compared with those of SP3, and in comparisons between FP4 and SP4, 2,862 and 1,308 genes were up‐ and downregulated, respectively, (Figure S4A; Table S3). Among the 2,842 FP3‐enriched genes (i.e., those upregulated in FP3 relative to SP3), 147 were associated with an FP3‐enriched dTHS, 455 were associated with a SP3‐enriched dTHS, and 19 showed dTHSs in both. Among the 1,091 SP3‐enriched genes, 130 were associated with a SP3‐enriched dTHS, 47 were associated with a FP3‐enriched dTHS, and three showed dTHSs in both (Figure S4B). A similar pattern was also observed in the comparison between SP4 and FP4 (Figure S4B). Many gene ontology (GO) terms with a false discovery rate (FDR) less than 0.05 were identified from DEGs associated with dTHSs described among the FP3‐enriched genes, many of the 147 genes associated with FP3‐enriched dTHSs were involved in transcription regulator activity, while many of the 455 genes associated with SP3‐enriched dTHSs mainly participated in various response pathways. However, the SP3‐enriched genes associated with both FP3‐enriched dTHSs and SP3‐enriched dTHSs were predominantly involved in some basic biological developmental events such as the cell cycle, DNA metabolic processes and the nucleus (Figure S4B).

These results indicate that approximately 20% of DEGs in SP3 *versus* FP3 and approximately 10% of DEGs in SP4 *versus* FP4 were associated with dTHSs and could be regulated at the transcription regulatory level along with changes in chromatin accessibility. In addition, the expression of a gene associated with a dTHS might be activated or repressed due to the function of TFs that bind to THSs. The activated genes of WXS(F) that are primarily involved in defense responses might be repressed or not activated in WXS(S), while the activated genes of WXS(S) mainly correlated with basic developmental processes might be partly repressed in WXS(F) (Figure S4B). This phenomenon indicates that WXS(F), which suffered an alteration of temperature, can activate temperature‐responsive mechanisms that reestablish homeostasis and protect normal development of the anther.

### Multiple transcriptional regulatory processes during fertility conversion identified by global transcriptome analysis

The young panicles of WXS(F) and WXS(S) at P3 and P4 showed obviously cytological differences (1E–R), so their global transcriptome profiles were examined to investigate key biological processes during fertility conversion. We identified six clusters (designated C1–C6) with specific expression patterns by analysis of all 7,870 DEGs (3A; Table S4). In C2 and C6, the genes were gradually downregulated from P3 to P4, with higher expression in WXS(S) (C2) or in WXS(F) (C6) (3A). In the other clusters, gene expression relatively increased from P3 to P4 with higher expression in WXS(F) (C1, C3, C5) or WXS(S) (C4) (3A).

**Figure 3:**
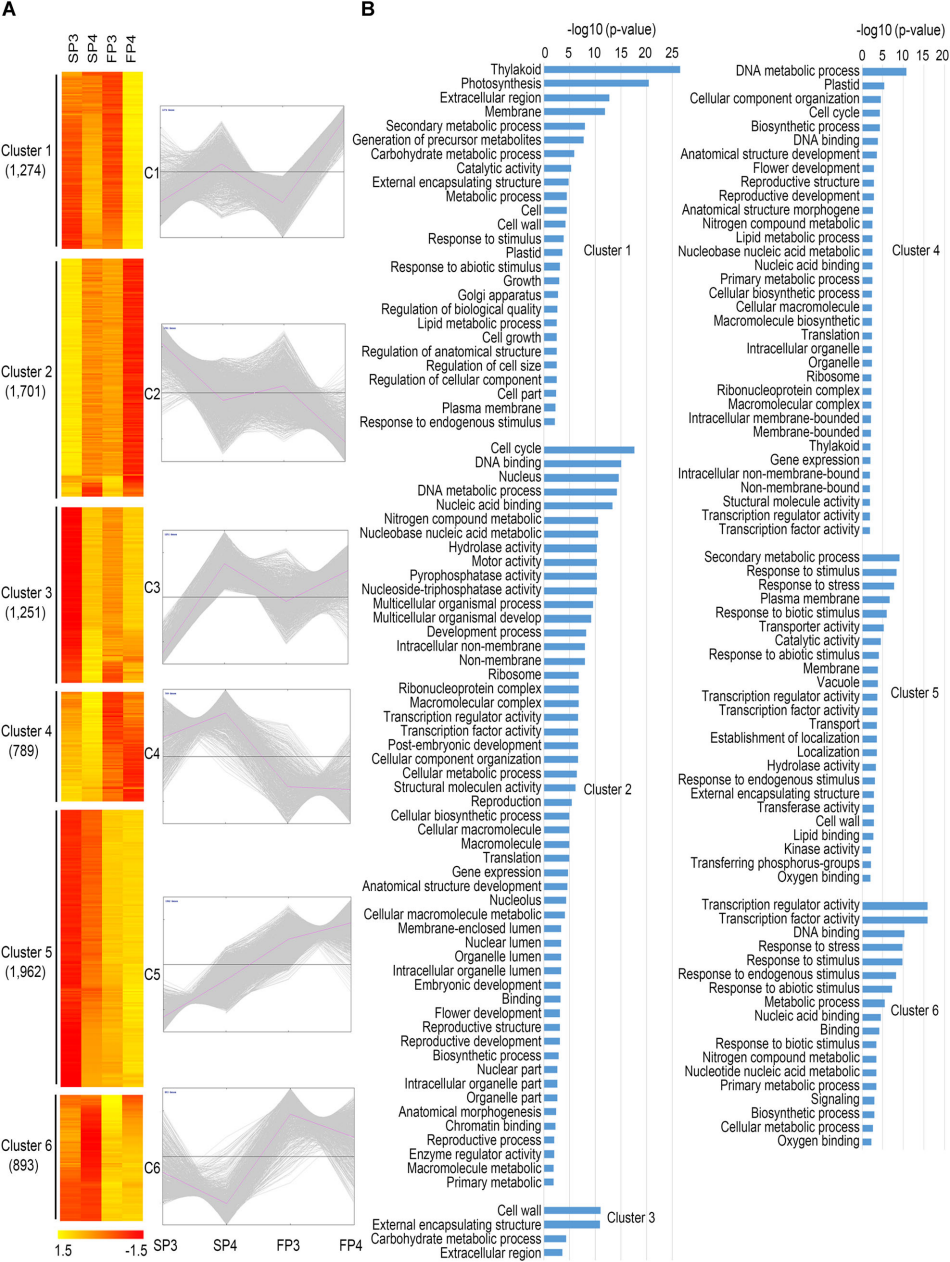
**Genome‐wide gene expression profiles of all differentially expressed genes (DEGs) from all four panicle tissue samples** **(A)** Heat map of all 7,870 DEGs classified into six distinct patterns (clusters 1–6) by the K‐means clustering method. Expression values are scaled per gene across samples. Diagrams of the expression patterns are shown (right), and the number of genes in each cluster is indicated. **(B)** Enriched gene ontology (GO) process terms for clusters 1 to 6 (from **A**), which were summarized using agriGO. The length of each bar indicates the overrepresentation significance of a group of GO terms.

To elucidate the functional implications of the clustered DEGs, the GO terms of genes that were preferentially expressed at P3 (C2 and C6) were mainly related to DNA binding, DNA metabolic processes and TF activity, which was consistent with the function of the panicle during meiosis (3B; Table S5). In addition to the basic cellular processes identified at P3, genes with higher expression in WXS(S) were predicted to mainly participate in the cell cycle (C2), while those with higher expression in WXS(F) were primarily involved in TF activity and response to stress (C6), which might indicate that fertility conversion could be regulated by TFs in response to temperature fluctuation. At P4, the DEGs with higher expression in WXS(S) were still mainly involved in the DNA metabolic process and cell cycle (C4); conversely, the DEGs with higher expression in WXS(F) were enriched in photosynthesis, carbohydrate metabolic processes, cell wall and stress response (C1, C3, C5) (3B; Table S5). These results suggest that genes preferentially expressed in WXS(S) mainly participate in basic cellular developmental activities that would ensure normal growth, potentially at the cost of delayed anther development. However, genes preferentially expressed in WXS(F) represent numerous TFs that may play vital roles in modulating and fine‐tuning the transcriptional program in response to temperature stimuli during the anther development stage.

**Figure 4:**
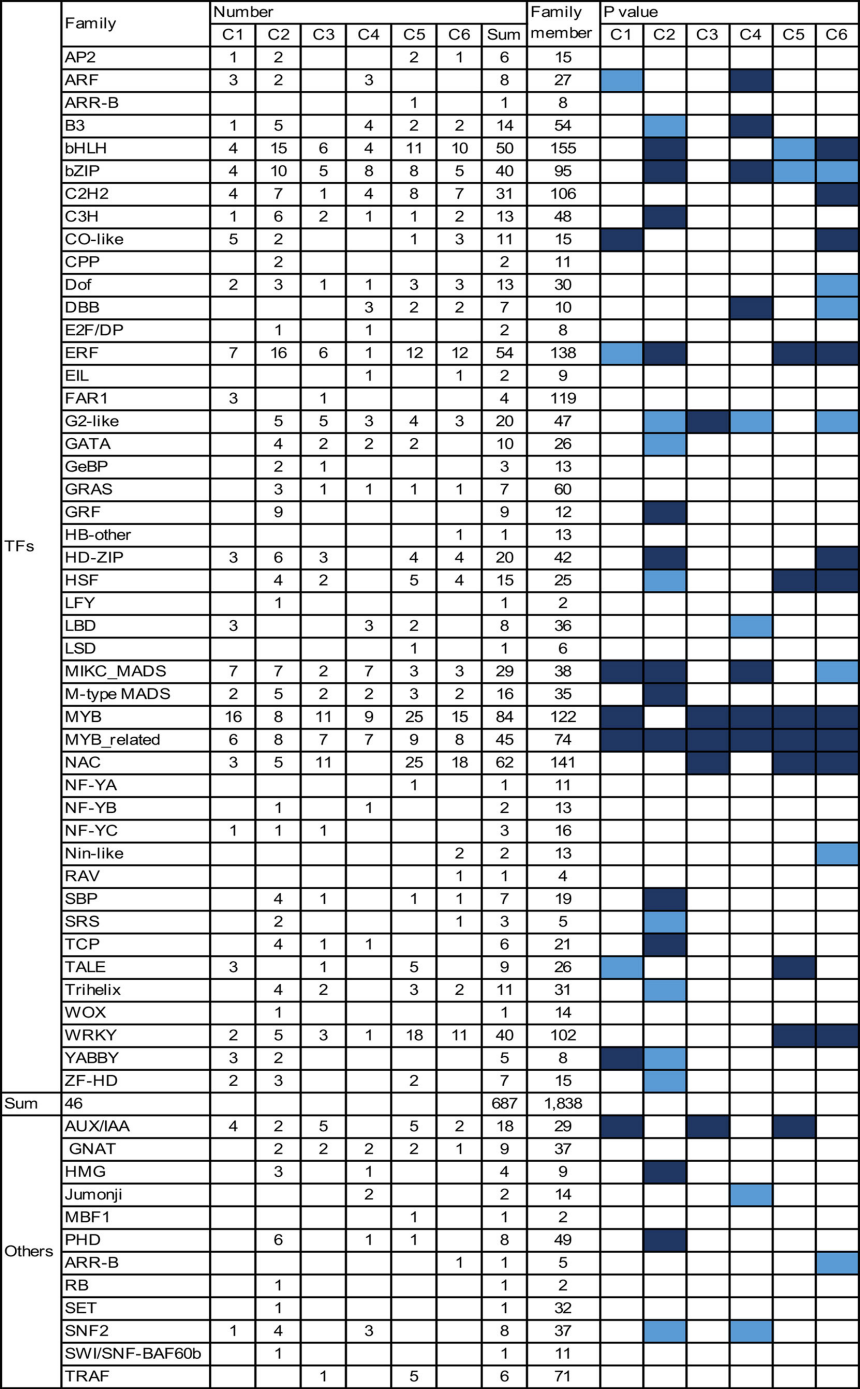
**Expression profiles of differentially expressed genes (DEGs) encoding transcription factors (TFs) during fertility conversion** Numbers of DEGs and enrichment of a given gene family encoding TFs are shown. Gene members corresponding to different expression patterns (clusters 1–6, as described in 3A) or members of a dedicated gene family are indicated. Enrichment of the gene family was determined by calculating the *P*‐values by Fisher's exact test.

To identify the key candidate transcriptional regulators involved in fertility conversion, the expression profiles of TF genes were analyzed in detail. A total of 687 TF genes belonging to 46 families were differentially expressed with distinct patterns (Figure 4). Among these TF families, the GRF, M‐type MADS, SBP and TCP members, which have been reported to regulate floral organ morphogenesis, pollen development, cell cycle, and hormone signaling, respectively (Ryu et al. 2009; Zhang et al. 2018), mainly exhibited a C2 pattern (higher expression in SP3) and might mainly regulate cell cycle and diverse developmental processes. Most of the DEGs encoding MYB and NAC proteins, two large families that regulate secondary wall biosynthesis (Shen et al. 2009; Zhong et al. 2011), were significantly enriched for genes expressing the C5 and C6 patterns (higher in WXS(F)), suggesting that the temperature stimuli might activate temperature‐dependent responses and cell wall synthesis in WXS, similar to the microRNA‐dependent regulatory pathways that participate in WXS fertility conversion suggested by Zhang et al. (2016). It has been shown that miR156‐to‐SPL, miR159‐to‐GAMYB, and miR164‐to‐NAC regulatory networks can regulate certain morphological changes and developmental and metabolic activities that influence fertility conversion (Zhang et al. 2016).

Our qRT‐PCR results also indicated that out of 13 randomly selected genes and *tms5*, *Ub*_*L40*_ *1*, *Ub*_*L40*_ *2*, and *Ub*_*L40*_ *4*, all 17 genes showed the same expression patterns between the relative expression level in qRT‐PCR and fold changes in RNA‐seq data (Figure S5A). Meanwhile, a hybridization experiment and genetic analysis also showed that the male sterility gene of WXS was a *tms5* mutant allele (Figure S5B, C). In the present study, the *tms5* kept mutant with defective RNase Z^S1^ in both WXS(F) and WXS(S). The expression of *tms5* (*LOC_Os02g12290*) showed no obvious difference between WXS(F) and WXS(S), indicating that expression of *tms5* itself was temperature‐insensitive (Figure S5A). However, genes encoding members of the conserved ubiquitin‐60S ribosomal protein L40 family (*Ub*_*L40*_), namely, *Ub*_*L40*_ *1* (*LOC_Os09g27930*), *Ub*_*L40*_ *2* (*LOC_Os03g15370*), and *Ub*_*L40*_ *4* (*LOC_Os09g31031*), showed significantly higher expression levels in WXS(S) than in WXS(F) (Figure S5A), which was consistent with the phenomenon that high temperatures induce the accumulation of *Ub*_*L40*_ mRNAs in *tms5* mutants, causing defective pollen production and male sterility (Zhou et al. 2014). Further, combined with the accessible chromatin regions of the *Ub*_*L40*_ family and *tms5*, *Ub*_*L40*_ *1*, and *Ub*_*L40*_ *4* had an obviously unique THS in FP3, while *Ub*_*L40*_ *2* and *tms5* had relatively similar chromatin accessibility patterns between WXS(F) and WXS(S), indicating that *Ub*_*L40*_ mRNA expression might be regulated by key TFs that play a vital role in fertility conversion (Figure S6A).

### ERF141/MADS7/MADS50/MYB modules affect various metabolic pathway‐related genes to control anther and pollen development

Fertility conversion of WXS is a complex process that is controlled not only by *Ub*_*L40*_ mRNA expression but also by related transcription regulatory processes that can affect pollen development. Therefore, the role of transcriptional regulation in fertility conversion is worthy of special focus to determine the regulatory modules. As previously described, THSs represent nucleosome‐free and accessible chromatin regions occupied by various TFs that could regulate the expression of nearby genes. To reveal the TF regulatory networks and specific TFs involved in fertility conversion of WXS, we used MEME‐ChIP analysis (Machanick and Bailey 2011) to identify key TFs and identify overrepresented motifs of known TFs in the differentially accessible regions of each sample. A total of 80, 88, 57, and 49 overrepresented motifs were identified within the FP3‐, SP3‐, FP4‐ and SP4‐enriched dTHSs, respectively, using pairwise comparisons of SP3 *versus* FP3 and SP4 *versus* FP4 (Table S6). Next, to narrow our list of candidate TFs to be investigated, the DEGs in our RNA‐seq data were used to determine which TF genes showed differential expression (Table S3). In total, 3, 13, 8, and 5 TF genes that had at least a two‐fold difference in their relative expression in FP3, SP3, FP4, and SP4, respectively, were separately identified for further investigation (5A, 5B, S6B, C; Table S7). In FP3, only genes for ERF TF family members were enriched, while genes for various TFs were enriched in SP3, including GATA10, ERF141, MADS7, and MADS50.

**Figure 5:**
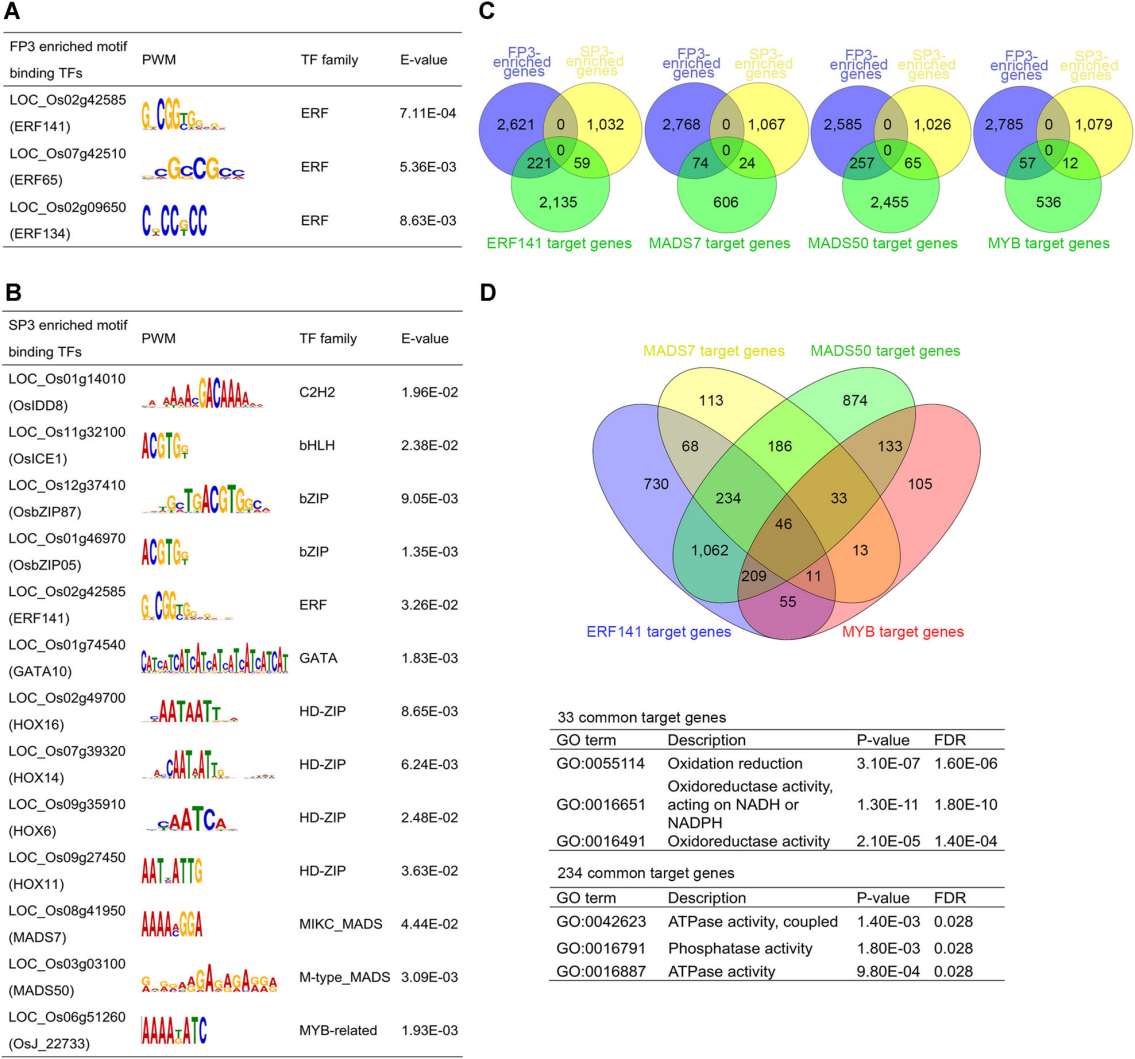
**Predicted sequence motifs and target genes for WXS‐enriched transcription factors (TFs) in meiosis (P3)** **(A)** FP3‐enriched Tn5 transposase hypersensitive site (THS) sequences and **(B)** SP3‐enriched THS sequences were analyzed with MEME‐ChIP. Motifs that had an E‐value equal to or less than 0.05 were considered significant. Only those transcription factor (TF) genes that had at least a two‐fold higher expression difference among all the above differentially expressed genes (DEGs) were retained. Position weight matrix (PWM) described the probability to find the respective nucleotides A, C, G, T on each position of a motif. **(C)** Venn diagrams showing target genes for ERF141, MADS7, MADS50, and MYB and their overlap with FP3‐enriched genes and SP3‐enriched genes. **(D)** Overlap of ERF141, MADS7, MADS50, and MYB target genes. Gene Ontology (GO) analysis was performed to illustrate the biological functions of co‐targeted genes. The upper table shows significantly enriched GO terms for all 33 genes targeted by MADS7, MADS50, and MYB. The lower table lists significantly enriched GO terms for the 234 genes co‐targeted by ERF141, MADS7, and MADS50.

Subsequently, we analyzed dTHSs from rice panicles using find individual motif occurrences (FIMO) to identify THSs that contained a motif for the TFs of interest, which were thus considered to be predicted TF binding sites. These predicted binding sites were then localized to the nearest TSS to identify the putative target genes for each TF (5A, B; S6B, C; Tables S8, S9). For further analyses, we first focused on four representative TFs (ERF141, MADS7, MADS50, and MYB) that were enriched in SP3. Using the aforementioned methods, we discovered 2,415 predicted target genes for ERF141, 704 for MADS7, 2,777 for MADS50, and 605 for MYB (5C; Table S8). By comparing these target genes with DEGs, we observed that a large percentage of the total target genes of all four TFs were FP3‐enriched genes (9%–11%), while some others were SP3‐enriched genes (2%–3%) (5C). Therefore, these TFs with dual function (i.e., associated with genes upregulated in both SP3 and FP3) might be primarily context‐dependent or temperature‐dependent, meaning that the actual function of the TF mainly relies on other surrounding factors and temperature fluctuations (Ikeda et al. 2009; Li et al. 2016). Each of the four SP3‐enriched TFs might generally serve as activators for SP3‐enriched genes, but might also have context‐dependent repression functions for FP3‐enriched genes involved in stress response and TF activity. Next, we examined the potential function of the co‐regulatory relationship of the four TFs, which had 46 genes as common targets (5D). A STRING network of protein interactions among these 46 target genes showed a correlation between cytochrome P450 and aldehyde oxidase, which might be repressed or not activated by MADS, MYB and ERF co‐regulation. Some of these target genes have been shown to be essential for anther cuticle and pollen exine formation in rice, likely resulting in male sterility in WXS(S) (Figure S6D).

### GATA10 regulates fertility conversion *via* modulation of *Ub*_*L40*_ expression

The RNase Z^S1^ encoded by *TMS5* processes the mRNAs of three *Ub*_*L40*_ genes into multiple fragments *in vitro* and *in vivo* (Zhou et al. 2014). In *tms5* mutants, high temperatures induce the accumulation of *Ub*_*L40*_ mRNAs in MMCs, causing defective pollen production and male sterility (Zhou et al. 2014). This finding highlights the impact of temperature on RNA metabolism at the post‐transcriptional level and the role of temperature‐dependent mRNA splicing, which may be harmful to the cells in the developing anther, leading to male sterility. However, the reason why *Ub*_*L40*_ mRNA can be overaccumulated under high‐temperature conditions at the transcriptional regulatory level has been unclear. In the present study, we speculated that some of the SP3‐enriched TFs mentioned above might regulate the expression of *Ub*_*L40*_ mRNA and affect fertility conversion. To test this hypothesis, we conducted dual‐luciferase system analysis to test the interactions of GATA10, ERF141, MASD7, MSDS50, HOX16, and MYB with the promoters of *Ub*_*L40*_ *1*, *Ub*_*L40*_ *2*, and *Ub*_*L40*_ *4*. This result indicated that only GATA10 could bind to the promoters of *Ub*_*L40*_ *2* and *Ub*_*L40*_ *4* and modulate their expression (6A). The subcellular localization result showed that in WXS protoplasts, the fluorescence signal of the GATA10‐HBT fusion vector overlapped with that of bZIP63‐RFP, a marker for the nucleus (6B). This result suggested that GATA10 was located in the nucleus, consistent with its role as a TF.

**Figure 6:**
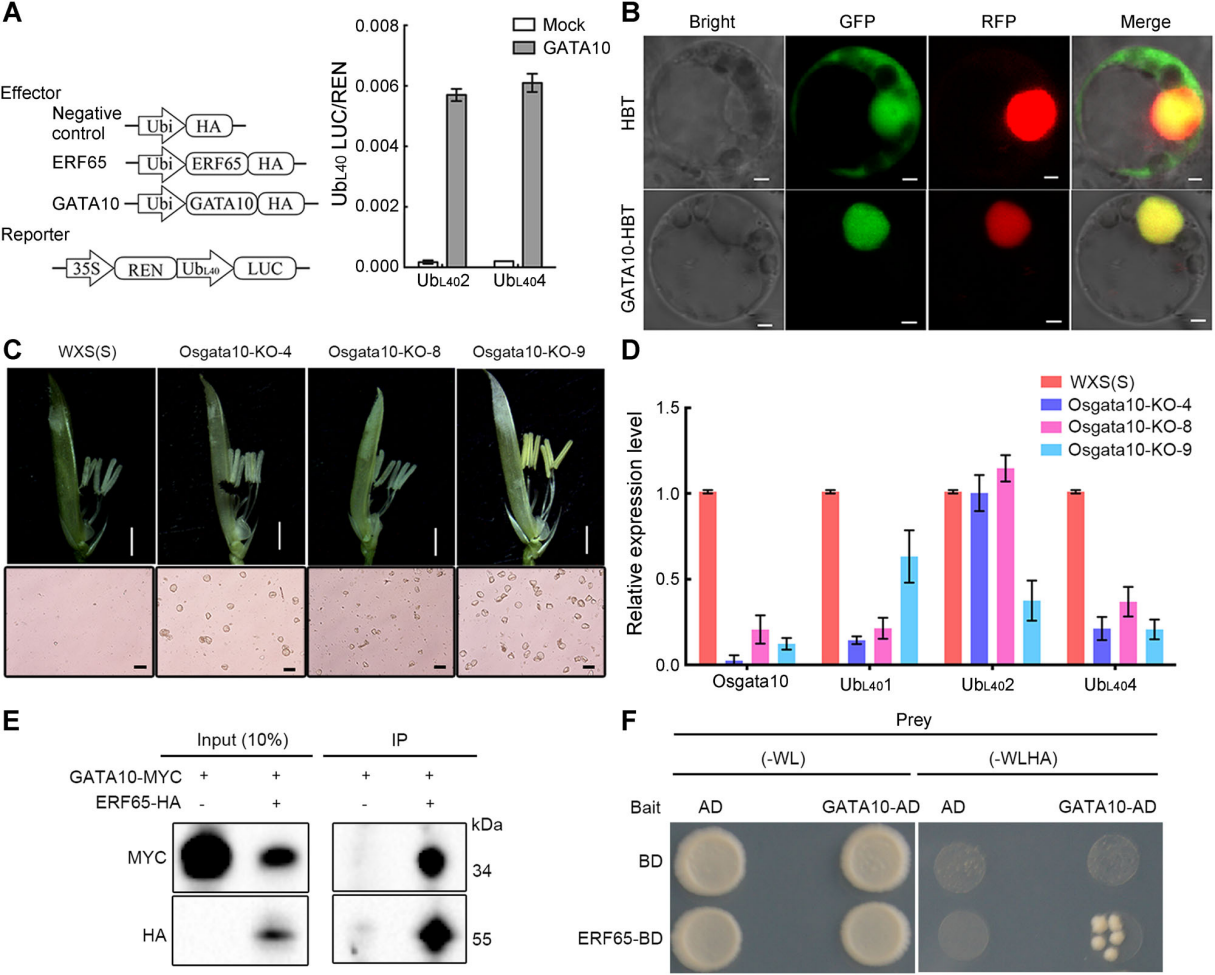
**Functional analysis of *Osgata10* with regulation of *Ub*_*L40*_ messenger (mRNA) expression** **(A)** Schematic diagrams of the effector and reporter plasmids used in the dual‐luciferase assay. The 35S:REN‐Ub_L40_Pro:LUC reporter constructs were transiently expressed in rice protoplasts together with a control vector (Mock) or TF‐gene‐containing effector vector. LUC, firefly luciferase; REN, Renilla luciferase. Error bars indicate *SD* (*n* = 5). **(B)** Subcellular colocalization of transiently expressed HBT (as control) or GATA10‐HBT with a nuclear marker (bZIP63‐RFP) in WXS protoplasts. Bars = 2 µm. **(C)** Anther morphology and pollen fertility comparisons of *Osgata10*‐KO‐4, *Osgata10*‐KO‐8, *Osgata10*‐KO‐9 and WXS(S) plants at the restrictive temperature. The anthers of the *Osgata10*‐KO transgenic plants turned yellow and contained pollen. Scale bars: 1 mm (top) and 5 µm (bottom). **(D)** The relative expression levels of *Osgata10, Ub*_*L40*_ *1, Ub*_*L40*_ *2, and Ub*_*L40*_ *4* were determined by quantitative RT‐PCR in WXS(S) (as control) and *Osgata10*‐KO transgenic plants at the restrictive temperature. Bar = means ±  *SD* from three biological repeats. **(E)** The interaction between GATA10 and ERF65 in a CoIP assay. GATA10‐MYC was transiently expressed in rice protoplasts together with ERF65‐HA. The protein complex in *in vivo* CoIP assays was precipitated using an anti‐HA antibody and then detected by western blot using an anti‐MYC antibody. Ten percent of input protein served as a control. **(F)** ERF65 interacts with GATA10 in yeast two‐hybrid assays. Yeast cells were grown on synthetic dropout medium lacking Leu and Trp (‐WL) as a control, and on medium lacking Leu, Trp, His, and adenine (‐WLHA) to stringently select for positive interactions.

To confirm whether GATA10 could regulate fertility conversion by modulating the expression of *Ub*_*L40*_, we designed two independent targets within *Osgata10* (Figure S7A) and used the CRISPR‐Cas9 system to disrupt *Osgata10* in the WXS background (designated *Osgata10*‐KO). We used these mutants for the phenotypic analysis. At the restrictive temperature (>22°C), the anthers of the *Osgata10*‐KO lines turned yellow, similar to normal anthers, and the pollen appeared and could be stained by I_2_‐KI but was shrunken and misshapen (6C); in contrast, WXS(S) showed abnormal anthers and non‐pollen type of sterility. Subsequently, we performed qPCR analysis, and the *Osgata10*‐KO plants exhibited significantly reduced levels of *Ub*_*L40*_ *1* and *Ub*_*L40*_ *4* mRNAs (6D). However, no detectable differences were observed in the *Osgata10*‐overexpressing WXS transgenic plants (designated *Osgata10*‐OE) compared with WXS(F) at the permissive temperature (≤22°C) (Figure S7B). Therefore, the overaccumulation of GATA10 was not enough to improve its ability to regulate transcription of nearby target genes without accessible chromatin regions. These results indicate that the loss of function of *Osgata10* results in some pollen production and low expression of *Ub*_*L40*_ mRNAs and revealed that GATA10 regulates fertility conversion at the transcription regulatory level *via* modulation of *Ub*_*L40*_ expression.

Moreover, GATA10 targeted a large number of genes annotated with the GO terms of amino acid binding and carboxylic acid binding, consistent with its known function in RNA polymerase II TF binding. This observation indicated that the interaction of TF‐to‐motifs or even TF‐to‐TF was likely mediated by GATA10 (Figure S7C; Table S8). To test whether GATA10 interacted with other dTHS‐enriched TFs, we performed co‐immunoprecipitation (CoIP) assays using fusion proteins transiently expressed in rice WXS protoplasts. The results of the CoIP assays showed that GATA10 interacted with ERF65 (6E); furthermore, yeast two‐hybrid (Y2H) assays also indicated that GATA10 directly interacted with ERF65 (6F). These results revealed that GATA10 interacts with ERF65 to mediate transcriptional regulation.

Taken together, our study reveals that these ERF141/MADS7/MADS50/MYB modules directly or indirectly affect various metabolic pathway‐related genes that control anther and pollen development, and furthermore, that GATA10 is a key component that regulates fertility conversion in *tms5* mutants by modulating the expression of *Ub*_*L40*_ mRNAs.

## DISCUSSION

### Accessible chromatin profiling is fundamental for revealing the regulatory functions of transcription factors

The PTGMS rice line WXS displays predominantly temperature‐controlled conversion from male sterility to fertility, with little effect of photoperiod. It is male‐sterile when anther development occurs at temperatures higher than 22°C and male‐fertile when the temperature is in the range of approximately 21–22°C (Zhou et al. 2012, 2014; Zhang et al. 2016). Due to unpredictable alterations of environmental temperature, failure to produce hybrid rice seeds frequently occurs in PTGMS lines in the field. The PTGMS gene in WXS has been shown to be *tms5* (Figure S5B, C), but little was previously known about the transcriptional regulatory switches responsible for genome‐wide gene expression in response to temperature fluctuation that might have an impact on fertility conversion (Wang et al. 2003).

Male reproduction in plants is very sensitive to environmental alterations. In the present study, obvious cytological differences between WXS(S) and WXS(F) were observed in anther transverse sections from different panicle developmental periods (1E–R). RNase Z^S1^, encoded by *TMS5*, processes the mRNAs of three *Ub*_*L40*_ genes into multiple fragments both *in vitro* and *in vivo* (Zhou et al. 2014). In *tms5* mutants, high temperature induces accumulation of *Ub*_*L40*_ mRNAs in MMCs, causing defective pollen production and male sterility (Zhou et al. 2014). This finding highlights the impact of temperature on RNA metabolism at the post‐transcriptional level and the role of temperature‐dependent mRNA splicing, which may be harmful to the cells in anther development and lead to male sterility. In the present study, the *tms5* kept mutant with defective RNase Z^S1^ in both WXS(F) and WXS(S), and we inferred that GATA10 regulated fertility conversion by modulating the expression of *Ub*_*L40*_ at the transcription regulatory level. Collectively, the ability of *Ub*_*L40*_ mRNA to accumulate at the high temperature might not only depend on mRNA decay at the post‐transcriptional level, but also partly on transcriptional regulation by various temperature‐dependent TFs.

Gene expression is not only controlled by a specific promoter, but diverse TFs and epigenetic mechanisms also influence final gene expression (Sullivan et al. 2014). Therefore, ATAC‐seq was performed to investigate the similarities and differences in accessible chromatin landscapes in WXS(F) and WXS(S), allowing us to identify and analyze TFs that act specifically in one sample type versus the other at the same stage (Buenrostro et al. 2013, 2015). Since the majority of THSs were similar in WXS(F) and WXS(S), we performed a quantitative analysis to identify THSs that were differentially accessible between WXS(F) and WXS(S) at P3 and P4 (Table S1). WXS(S) obviously had many more highly accessible chromatin regions than WXS(F), which were then assigned to their nearest TSS as putative target genes regulated by the dTHSs (Table S2). The numbers, locations, and levels of Tn5 transposase sensitivity of THS sites can vary significantly among different sample types and may be induced by temperature fluctuations. Thus, the identification of key regulatory modules under different temperatures will be an essential future effort to achieve a comprehensive understanding of the transcriptional regulation of gene expression during fertility conversion. When compared with DEGs, the expression of these target genes might be activated or repressed due to the function of TFs that bind to the THSs. Most of the target genes associated with WXS(S)‐enriched‐dTHSs were repressed, and they might be activated (or not repressed) in WXS(F) in response to temperature. GO analysis of these genes revealed known functions in response to the altered environment, which indicated that WXS(F) suffering from temperature alterations activated temperature‐responsive mechanisms that reestablished homeostasis and protected normal anther development (Figure S4B).

### Multiple biological processes, especially cell wall formation, play vital roles in fertility conversion

To further investigate multiple key biological processes during fertility conversion, the DEGs were clustered into six clusters (3A). We confirmed that the DEGs that were highly expressed in WXS(S) mainly participated in basic cellular developmental activities that would ensure normal growth, potentially at the cost of delayed anther development for energy conservation; however, the DEGs that were highly expressed in WXS(F) encoded various TFs that might play vital roles in modulating and fine‐tuning the transcriptional program in response to temperature stimuli during anther development, leading to male fertility (3B). When we focused on identifying key candidate regulators involved in fertility conversion, genes for various TFs, such as GRF, M‐type MADS, SBP, and TCP, were observed to be enriched in WXS(S)‐P3, which might mainly regulate the cell cycle and diverse basic developmental processes. In addition, genes for NAC and MYB, two large families regulating secondary wall biosynthesis, were significantly enriched in WXS(F), suggesting that temperature stimuli may activate stress responses and cell wall synthesis in WXS (Figure 4).

During pollen development, the anther wall not only forms a protective covering for the developing microspore, but the innermost layer of the wall (i.e., the tapetum) also provides nutrition to them (Shi et al. 2015). The anther cuticle and pollen exine are regarded as two main protective barriers and initial sensors that immediately respond to environmental challenges, and structural modifications of the cell wall are essential during defense responses against temperature fluctuations (Thoma et al. 1994). The initial signal of changes in temperature would trigger downstream transcription regulation and signal transduction, stimulating temperature‐responsive mechanisms that maintain homeostasis and protect and repair damaged cellular membranes and proteins. Consistent with this mechanism, many DEGs enriched in WXS(F) were primarily involved in defense responses and secondary metabolic processes, potentially to redistribute energy resources for pollen development. Additionally, genes for diverse kinases were enriched in WXS(F) (Figure S8). However, the DEGs without initial signal stimuli enriched in WXS(S) mainly participated in basic biological developmental processes, such as DNA metabolic process and cell cycle, which might suppress unnecessary defense responses and thus maintain growth and energy homeostasis (3B). Generally, constitutive activation of defense responses consumes considerable cellular energy, which might occur at the cost of plant growth and development. Moreover, the most sensitive stress‐responsive mechanism is photosynthesis, to which carbohydrate metabolism is directly linked (Casal and Questa 2018).

Secondary metabolites affect pollen developmental processes such as the deposition of lipid, which is required for proper pollen wall formation, and their biosynthesis is tightly regulated by the developmental stage, tissue or several stress situations (Thoma et al. 1994). For instance, cyp703a2 mutants in *Arabidopsis* produce partially sterile pollen grains displaying an abnormal exine with no obvious sporopollenin deposition (Morant et al. 2007). In this study, KEGG analysis of DEGs in WXS(S) versus WXS(F) revealed a principal enrichment of genes related to phenolic metabolites, such as phenylpropanoid biosynthesis and phenylalanine metabolism (Figure S9A, B). Phenolic metabolites are parts of the chemical composition of sporopollenin, a major component of the tough exine walls of microspores and pollen grains (Ariizumi and Toriyama 2011). In addition, cytological observations of the young panicles of WXS(F) and WXS(S) also showed significant differences in the development of the anther tapetum and pollen exine (1E–R).

### ERF141/MADS7/MADS50/MYB modules affect metabolic pathway‐related genes to control anther and pollen development

In a search for specific TFs that might be responsible for fertility conversion, we analyzed differentially enriched THS regions to identify putative *cis*‐regulatory elements as well as the TFs that bind them. Compared with the DEGs from our data, we identified various TF genes that were differentially expressed in each sample and identified their nearby target genes (5A, B; S6B, C). Among these TFs, members of the ERF, MADS, MYB, and NAC families have been well characterized for their regulatory roles in response to abiotic stress, plant morphology, and secondary wall biosynthesis (Shen et al. 2009; Zhong et al. 2011; Kobayashi et al. 2012), which might be responsible for fertility conversion. For instance, our target NAC members are reported to function in plant adaptation to temperature fluctuation, likely through transcriptional reprogramming of downstream temperature‐sensitive genes, and some of them regulate cell wall development in relation to lignocellulosic bioenergy production (Shen et al. 2009). The expression of rice secondary wall‐specific cellulose synthase genes is regulated by OsMYB86‐L1 or other R2R3‐MYB TFs (Noda et al. 2015). As a floral organ identity gene, OsMADS7 has been shown to participate in stabilizing rice amylose content at high temperatures, and OsMADS50 has used been in a model for the photoperiodic regulation of rice flowering, which consists of both suppression and activation pathways (Lee et al. 2004; Ryu et al. 2009; Zhang et al. 2018). Members of the ERF family are implicated in plant defense programs, and they are also known to play roles as active repressors: these TFs contain an independent repressor domain that represses transcription directly by chromatin modifications such as histone methylation or deacetylation (Ohta et al. 2001). The same ERF TFs were enriched in both WXS(F) and WXS(S), which targeted different accessible chromatin regions with different target genes. The dual‐function TFs, such as the ERF family, are part of a functional switch that might involve direct mechanisms such as the changes in chromatin structure correlated with histone modification, changes in *cis* elements occupied by TFs, structural alterations of TFs caused by alternative splicing or post‐translational modifications, or functional alterations triggered by partnering with a specific TF or chromatin‐modifying complex (Ohta et al. 2001). Most of the above changes can be achieved by temperature fluctuation. These results are relevant to the observation that genes that are differentially expressed in various environmental situations may be associated with different transcription regulators, depending on whether the genes are activated or repressed.

### Transcriptional regulatory mechanisms of fertility conversion are mediated by GATA10 through the modulation of *Ub*_*L40*_ expression

**Figure 7:**
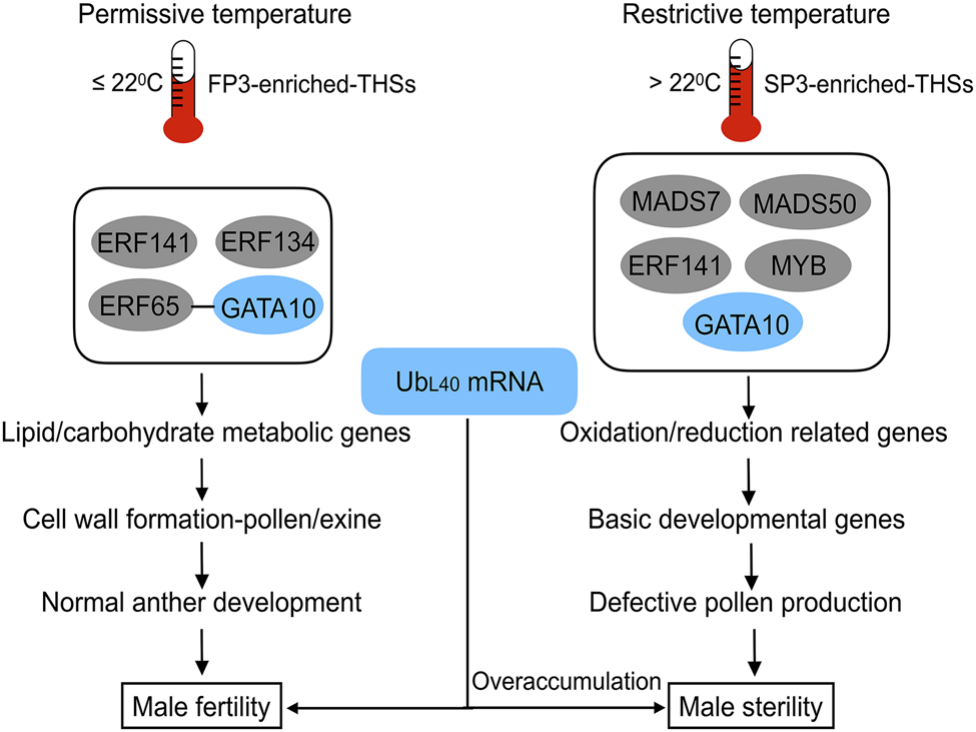
**An integrated view of the key transcriptional regulatory modules during fertility conversion** GATA10 regulates fertility conversion by directly modulating *Ub*_*L40*_ messenger RNA (mRNA) expression and by working as a mediator of the interaction with ERF65 or other transcription factors. The FP3‐ and SP3‐enriched transcription factors directly or indirectly regulate various metabolic pathway‐related genes to control anther and pollen development, which have been proven to cause fertility conversion. THSs, Tn5 transposase hypersensitive sites.

Our findings proved that only approximately 20% of the DEGs in SP3 *versus* FP3 and approximately 10% of the DEGs in SP4 *versus* FP4 were associated with dTHSs and could be regulated at the transcription regulatory level along with changes in chromatin accessibility. The remaining 80%–90% of the DEGs without obvious chromatin accessibility differences may have other explanations for their differential expression. For instance, these DEGs may be regulated at the post‐transcriptional level, rather than at the chromatin accessibility level that we measured, through mechanisms such as lncRNA, microRNA and alternative splice regulation. Therefore, the effect of the transcriptional regulatory level on fertility conversion is limited. WXS(S) has a non‐pollen‐abortion type of sterility, and the overaccumulation of *Ub*_*L40*_ mRNAs under the restrictive temperature is harmful to the cells in anther development, resulting in male sterility. The loss of function of *Osgata10* resulted in production of pollen without starch deposition, and the *Osgata10* knockout transgenic plants exhibited significantly reduced levels of *Ub*_*L40*_ *1* and *Ub*_*L40*_ *4* mRNAs (6C, D). However, because insufficient chromatin regions that can be occupied by GATA10 are accessible at the permissive temperature, the *Osgata10*‐overexpressing transgenic plants showed no detectable differences from the WXS(F) plants (Figure S7B). Accessible chromatin profiling is fundamental for revealing the regulatory functions of TFs. We demonstrated that the SP3‐enriched TF GATA10 directly activated downstream *Ub*_*L40*_ mRNA expression, which partly explained why *Ub*_*L40*_ transcripts could overaccumulate under high temperature and complemented the transcriptional regulatory mechanism of *Ub*_*L40*_ mRNA regulation. Moreover, GATA10 acted as a mediator of the interaction with FP3‐enriched TF ERF65 (6E, F). It appears that the ERF141/MADS7/MADS50/MYB module affected metabolic processes in rice anthers that control anther and pollen development. These results are relevant to the finding that the ERF/GATA module represents an essential hub in downstream transcriptional regulation of fertility conversion. Furthermore, GATA10 is a key component for the regulation of fertility conversion through modulation of *Ub*_*L40*_ expression (Figure 7).

In summary, here we have outlined an approach for combining accessible chromatin profiling with gene expression analyses to understand transcriptional regulation and identify key new regulatory modules, especially the mediator GATA10, in response to temperature fluctuation. This information can provide important evidence regarding the transcriptional regulatory mechanisms of fertility conversion in two‐line hybrid rice.

## MATERIALS AND METHODS

### Plant materials and growth conditions

The rice PTGMS line WXS (*Oryza sativa* ssp. indica) was selected and bred by the State Key Laboratory of Hybrid Rice, College of Life Sciences, Wuhan University, Wuhan, China. We obtained the new plant variety right (CNA20120607.9) for WXS, which was granted by the Ministry of Agriculture of the People's Republic of China. WXS was grown in the paddy fields of Wuhan University (30°34′N; 114°17′E), Wuhan, China, in the summer of 2016. From July 20 to August 30, 2016, the average temperature was 29.7°C, with a 14‐h light/10‐h dark photoperiod, which caused WXS male sterility (WXS(S)). When the panicle length reached approximately 1 cm (before the stages of early premeiosis), half of the plants were transferred to growth chambers with an average temperature of 22°C (Zhou et al. 2014; Zhang et al. 2016) and a 14‐h light/10‐h dark photoperiod and 77% relative humidity; these conditions were close to the natural illumination levels, humidity values and other environment factors that could induce the conversion of WXS into male fertility (WXS(F)) (Zhou et al. 2012; Zhou et al. 2014). Anther developmental stages were evaluated based on anther length and cross‐section, and young panicles in the meiosis period (P3) and uninucleate period (P4) were collected from WXS(S) and WXS(F) (Zhang and Wilson 2009). Mature pollen grains were stained with 1% I_2_‐KI solution. Panicles from various stages of development were fixed with FAA solution at 4°C to generate transverse sections of anther. In addition, young panicles were collected, immediately frozen in liquid nitrogen, and ground into a fine powder for ChIP‐seq, ATAC‐seq, and RNA‐seq experiments.

### Vector construction and plant transformation

The pYLCRISPR/Cas9Pubi‐H vector was used to create independent homozygous *Osgata10*‐knockout (*Osgata10*‐KO) transgenic plants. Positive transgenic lines were grown at the restrictive temperature (>22°C). To generate *Osgata10*‐overexpressing plants (*Osgata10*‐OE), the coding sequence was amplified and inserted into the pCXUN vector driven by the ubiquitin promoter. When the panicle length reached approximately 1 cm, positive transgenic plants were transferred to growth chambers with an average temperature of 22°C. The young panicles of positive transgenic plants were collected, and pollen grains were stained in 1% I_2_‐KI solution for fertility observation.

### RNA isolation and gene expression analysis

Total RNA was extracted from *Osgata10*‐KO and *Osgata10*‐OE transgenic lines using TRIZOL reagent (Invitrogen, Waltham, MA, USA). Gene expression was investigated by qRT‐PCR. The actin gene was used as an internal standard and qRT‐PCR was performed with three repeats per gene. The primers used for qRT‐PCR are listed in Table S10.

### ATAC‐Seq library preparation and data processing

Intact nuclei were isolated using the Plant Nuclei Isolation/Extraction Kit (SIGMA, St. Louis, MO, USA). Briefly, finely powdered tissue was suspended in Nuclei Isolation Buffer (NIB), 0.3% TRITON X‐100 solution was added for cell membrane lysis, and 2.3 M sucrose solution was added for the preparation of intact nuclei. DAPI staining was applied for quality control. The chromatin of intact nuclei was fragmented and tagged following the standard ATAC‐seq protocol (Buenrostro et al. 2013, 2015). Two biological replicates were prepared for each tissue. Libraries were sequenced, with four libraries per lane, using standard methods for paired‐end 50‐bp reads, on an Illumina HiSeq. 4000 platform at Beijing Genomics Institute (BGI; http://www.genomics.cn).

We used Bowtie2 software to align the reads to *O. sativa* Nipponbare release 7 of the MSU Rice Genome Annotation Project reference genome and discarded reads that mapped to the mitochondrial or chloroplast genome (Langmead and Salzberg 2012). Only reads that aligned exactly once to the genome were used in the downstream analysis. Heat maps and average plots were generated using the “*computeMatrix*” “*plotHeatmap*”, and “*plotProfile*” functions in the deepTools package. The Pearson correlation coefficient was used to measure the relationship between the two biological replicates for each tissue. Peak calling was performed by MACS 1.4.2 with default parameters. The peaks called in this way were referred to as “transposase hypersensitive sites” or THSs. Integrative Genomics Viewer (IGV) 2.3.68 was used to visualize the WIG file. The differential analysis was performed using default settings by MAnorm (Shao et al. 2012). Only those THSs that had an |M‐value| greater than 1 were categorized as THS‐enriched in that sample type and were referred to as differential THSs (dTHSs). We mapped each dTHS to its nearest TSS using the PAVIS web tool and considered that to be the target gene.

### RNA‐seq and data analysis

All protocols were performed per the manufacturers’ instructions. Total RNA was extracted with RNAiso Plus (TAKARA, Japan). RNA quality was confirmed by gel electrophoresis. Contaminating DNA and rRNA were removed. Strand‐specific RNA‐seq libraries were synthesized using the NEBNext® Ultra^TM^ Directional RNA Library Prep Kit for Illumina® (NEB, Ipswich, MA, USA). The libraries were sequenced with the Illumina HiSeq X ten platform, and paired‐end reads were generated.

The reads were aligned to the MSU7 reference genome by HISAT2, discarding low‐quality alignments. Gene expression levels were calculated using StringTie by counting the numbers of reads mapped to each gene. The expression levels of genes were normalized to the fragments per kilobase per million read (FPKM) values. Three biological replicates per sample were averaged for the differential expression analysis. Differential expression analysis of the two samples was performed using the DESeq R package. Genes with an expression fold‐change >2 with a *P*‐value < 0.05 were classified as differentially expressed.

### Clustering analysis and GO analysis

All differentially expressed genes (DEGs) were normalized to the average expression values of the replicates. K‐means clustering with Euclidean distance was used to group genes based on merit analysis. Heat maps of gene expression values were generated in TreeView 1.1.6 or Microsoft Excel. To annotate the functions of the identified gene sets, the gene ontology information from agriGO was used. GO enrichment was derived using Fisher's exact test and a cut‐off FDR <0.05.

### Gene family enrichment analysis

Rice TFs and other transcriptional regulators were referenced to the plant TF databases PlnTFDB (http://plntfdb.bio.uni‐potsdam.de/v3.0/) and PlantTFDB (http://planttfdb.cbi.pku.edu.cn/). TFs and other transcriptional regulators were gathered from these two websites after discarding duplicates. Kinase genes were referenced to the Rice Kinase Database. Hypergeometric distribution analysis was performed to identify TF or kinase families enriched in the cluster‐grouped data sets.

### ChIP and ChIP‐seq data analysis

ChIP assays were performed using Anti‐H3K9ac and Anti‐H3K4me2 antibodies as previously described, with modifications (Li et al. 2008; He et al. 2010; Du et al. 2013). Chromatin was extracted from WXS panicles in isolation buffer and fragmented using micrococcal nuclease (NEB, Ipswich, MA, USA). ChIP‐ed DNA was submitted to the Beijing Genomics Institute (BGI; http://www.genomics.cn) for ChIP‐seq library construction and sequencing on the Illumina HiSeq. 2000 platform.

Over 20 million clean reads were aligned to the MSU7 reference using the SOAP2 aligner. SICER software was used to call histone modification peaks with default parameters (bandwidth, 200 bp; E‐value, 0.01). IGV was used to visualize the WIG file. The differential analysis was performed using default settings by MAnorm (Shao et al. 2012).

### Quantitative real‐time PCR for verification of RNA‐seq and ChIP‐seq

Quantitative real‐time polymerase chain reaction was performed with ChIP‐ed DNA to validate the ChIP‐seq results and with total RNA to validate the RNA‐seq results. Total RNA extraction and cDNA synthesis were performed as described above. Quantitative real‐time polymerase chain reaction analysis was performed using the FastStart Universal SYBR Green Master (Roche, Basel, Switzerland) on the ABI Step One Plus Real‐Time PCR system (Thermo Fisher Scientific, Waltham, MA, USA). The ChIP‐ed DNA level was calculated using the 2^−ΔΔCt^ method with the SuperArray ChIP‐qPCR data analysis template (Chakrabarti et al. 2002). Each reaction was carried out in triplicate. The rice actin gene was used as a reference gene for relative quantification. The primers used for qRT‐PCR are listed in Table S10.

### Transcription factor motif analysis

ATAC‐seq THSs were used for motif analysis, and masked sequences were run through MEME‐ChIP with default parameters to identify overrepresented binding motifs for known TFs in the differentially accessible regions of each sample (Machanick and Bailey 2011). The DREME, MEME, and CentriMo programs were used to identify overrepresented motifs, and Tomtom was applied to match these motifs to previously reported TF binding motifs. Motifs from both Cis‐BP and PlantTFDB databases were used in all motif searches, and only those that had an E‐value <0.05 were considered significant for further research.

### Defining target sites for transcription factors

We used FIMO (Grant et al. 2011) to identify motif occurrences for known TFs of interest; significant motif occurrences were considered to be those with a *P*‐value < 0.0001. Genome‐wide binding sites for a given TF were defined as THSs that also contained a significant motif occurrence for the factor.

### Protein interaction analysis using STRING

Gene lists were analyzed using the STRING database to identify the predicted interactions of target genes coregulated by TFs of interest. The network connections were visualized by their confidence score, where a thicker line indicates a higher interaction score. The genes were color‐coded by their clustering, as determined by the Markov Cluster Algorithm score set to 3.0. The minimum interaction threshold used in this study was set to 0.700. The inputs used for the STRING database were the *rice* gene IDs.

### Single nucleotide polymorphismsite sequencing and WXS testcross

In previous reports, TGMS lines, such as ANS‐1 and Zhu1S, have revealed a single nucleotide polymorphism (SNP) locus comprising a C‐to‐A transition at position 71 of *tms5* compared with the wild type AnnongN, which leads to the generation of a premature stop codon (Zhou et al. 2014). This SNP change was identical to the premature stop codon mutation mapped to the same gene (namely *ptgms2‐1*) in the non‐pollen type of PTGMS rice Guangzhan63S (Xu et al. 2011). Another SNP comprising a mutant site (C‐to‐G) in the PGMS gene *pms3*, was found in PGMS lines, such as NK58S and PA64S (Ding et al. 2012). To determine whether the two mutant SNPs exist in WXS, we selected three conventional rice varieties (93‐11, Nipponbare and Wuxiang B) and three two‐line male sterile lines (PA64S, NK58S, and Y58S) as controls to perform PCR amplification of the flanking regions of the SNPs. Then, the PCR products were cloned and sequenced. Next, to determine whether *tms5* or *pms3* is the major gene, WXS rice was separately crossed with PA64S and Y58S in Sanya Lingshui in winter (December, 2014 to March, 2015). Finally, the F_1_ seeds were obtained and planted in Wuhan in summer (May 2015 to August 2015). To analyze the pollen fertility of F_1_ plants, the mature anthers were also collected and stained with 1% potassium iodide solution (I_2_‐KI).

### Subcellular localization

The full‐length coding sequence of *Osgata10* was inserted behind the cauliflower mosaic virus (CaMV) 35S promoter into the HBT‐GFP vector to produce a 35S: *Osgata10*:GFP fusion vector for transient expression in rice WXS protoplasts by polyethylene glycol (PEG)‐mediated infiltration. The nucleus marker was bZIP63‐RFP. The empty HBT vector was used as a control. The GFP and RFP signals were visualized with a FV1000 confocal scanning microscope. The primers used for subcellular localization are listed in Table S10.

### Dual‐luciferase assay

For the dual‐luciferase assay, pGreenII 0800‐LUC vectors with LUC under the control of three Ub_L40_ promoters (1 kb upstream of ATG) were generated as reporters, with the *Renilla* luciferase (REN) gene under the control of the 35S promoter used as an internal control. To construct the overexpression vectors, the full‐length coding sequences of GATA10, ERF141, MASD7, MSDS50, HOX16, and MYB were amplified and cloned into the PCXUN vector; these constructs were used as the effectors. The empty PCXUN vector was used as the control for the effector. Rice shoot protoplasts were prepared and transfected using a PEG/calcium‐mediated method followed by an 18‐h incubation to allow transient expression. Firefly LUC and REN activities were measured with a Dual‐Luciferase Reporter assay kit (Promega, Madison, WI, USA). The LUC/REN ratios were calculated and three independent transformations were performed for each plasmid combination. All primers are listed in Table S10.

### Co‐immunoprecipitation assays

Co‐immunoprecipitation assays were performed as described previously (Yang et al. 2014). The full‐length coding sequence of ERF65 was separately amplified and cloned into the PCXUN vector fused with a HA tag, and GATA10 was cloned into PCXUN vector fused with a MYC tag. GATA10‐MYC and five other TFs with HA tags were coexpressed in rice protoplasts, and lysates were subjected to CoIP assays with anti‐HA antibodies (MBL, Japan). The precipitated proteins were detected by western blotting using an anti‐MYC antibody.

### Yeast two‐hybrid assays

Yeast two‐hybrid assays were performed according to the manufacturer's instructions (TAKARA, Japan). The full‐length coding sequence of ERF65 was cloned into pGBK‐T7 (BD), and full‐length coding sequence of GATA10 was cloned into pGAD‐T7 (AD). The vectors were then cotransformed into *Saccharomyces cerevisiae* strain AH109 and selected on SD/‐Leu/‐Trp plates for 3–4 days at 30°C. Protein–protein interactions were observed based on the growth of transformants on SD/‐Leu/‐Trp/‐His plates. Primers are listed in Table S10.

## AUTHOR CONTRIBUTIONS

Y.D. and J.J. designed the experiments; J.J. performed most of the experimental work and wrote the manuscript; J.J. and S.G. analyzed the data; Y.W. performed some western blot experiments; H.Z. provided the results of WXS testcrosses and SNP site sequencing; Z.Z. provided constructive advice regarding the experiments; Q.L. and Y.S. helped to perform plasmid extraction; H.C. and Y.Z. helped to collect rice WXS panicle materials; X.H. provided rice WXS seeds. All authors read and approved the paper.

## ACCESSION NUMBERS

The raw and processed ATAC‐seq, ChIP‐seq and RNA‐seq data described in this work have been deposited to the NCBI Gene Expression Omnibus (GEO) database under the record number GSE114090.

## Supporting information

Additional Supporting Information may be found qonline in the supporting information tab for this article: http://onlinelibrary.wiley.com/doi/10.1111/jipb.12871/suppinfo

**Figure S1.** ATAC‐seq profiling of nucleus quality, read alignments, fragment size distribution and correlation with published DNaseI hypersensitive sites
**(A)** Fluorescence microscope image of nuclei stained with the DNA‐binding dye DAPI (blue). Scale bar, 100 μm. **(B)** Mapped reads are all the reads that aligned to the *Oryza sativa* MSU7 reference genome. Nuclear Mapped Reads exclude the portion of reads that aligned to the mitochondrial or chloroplast genomes; % Aligned = Nuclear Mapped Reads/Clean Reads. Q20 and Q30 denote mapping quality scores. **(C)** Fragment sizes of ATAC‐seq reads for all four samples (FP3, FP4, SP3, SP4). The dotted line indicates the trendline. **(D)** Overlapping peaks of Tn5 transposase hypersensitive sites (THSs; this study) and published DHSs in rice. (**E**) The percent coverage of accessible chromatin regions occupying the rice genome.
**Figure S2.** Correlation of histone modification with Tn5 transposase hypersensitive sites (THSs) by ATAC‐seq
**(A)** Visualization by Integrative Genomics Viewer (IGV) showing enrichment of ATAC‐seq as well as ChIP‐seq in WXS(F) and WXS(S) at P3 and P4. Gene models are displayed on the bottom track. **(B)** Scatter plots comparing the enrichment between ATAC‐seq and ChIP‐seq. Pearson correlations of the reads per kilobase per million mapped reads (RPKM) values are shown. **(C)** The profile of H3K9ac and H3K4me2 marks (indicated by the number of ChIP‐seq reads) among genes with different expression levels based on our RNA‐seq data. The expressed genes were divided into five bins from lowest to highest expression.
**Figure S3.** Quantitative real‐time PCR validation of H3K9ac and H3K4me2 ChIP‐seq Fourteen genes were selected for the test (one with reduced, two with unchanged, and four with increased H3K9ac; five with reduced, one with unchanged, and one with increased H3K4me2 in ChIP‐seq, as shown on the left). Levels relative to the input are shown. The DNA level was calculated using the 2^−ΔΔCt^ method. Bar = means ± *SD* from three biological repeats.
**Figure S4.** The accessible chromatin regions with all differentially expressed genes (DEGs) from our RNA‐seq data
**(A)** DEGs based on pairwise comparison. Red dots represent upregulated genes, and green dots represent downregulated genes. **(B)** (Left) Venn diagram of overlap between FP3‐ or SP3‐enriched genes and genes associated with FP3‐ or SP3‐enriched differential Tn5 transposase hypersensitive sites (dTHSs). (Right) Venn diagram of overlap between FP4‐ or SP4‐enriched genes and genes associated with FP4‐ or SP4‐enriched dTHSs (right). Genes were considered “enriched” if they had a two‐fold or higher difference in expression between SP3 versus FP3 or SP4 versus FP4. GO analysis was performed to reveal the biological functions of overlapped genes.
**Figure S5.** Quantitative real‐time PCR verification of RNA‐seq results
**(A)** The relative expression levels of 17 genes were determined by quantitative RT‐PCR. Significant differences between the samples are indicated by ** (*P*‐value < 0.01) and * (*P*‐value < 0.05). Bar = means ± *SD* from three biological repeats. (**B**) WXS testcross with PA64S and Y58S. The mature anthers of F_1_ plants were collected and stained with 1% potassium iodide solution (I_2_‐KI). Scale bar: 2 mm (left); 20 μm (right). (**C**) The results of SNP site sequencing. Three conventional rice varieties (93‐11, Nipponbare and Wuxiang B) and three two‐line male sterile lines (PA64S, NK58S, and Y58S) as controls were used to perform PCR amplification of the flanking regions of the SNPs. Then, the PCR products were cloned and sequenced.
**Figure S6.** Predicted sequence motifs and target genes for WXS(F)‐ and WXS(S)‐enriched transcription factors (TFs) in the uninucleate period (P4)
(**A**) Accessible chromatin profiling of *Ub*_*L40*_ *1, Ub*_*L40*_ *2, Ub*_*L40*_ *4* and *tms5* in FP3, SP3, FP4 and SP4. (**B**) FP4‐enriched Tn5 transposase hypersensitive site (THS) sequences and (**C**) SP4‐enriched THS sequences were analyzed using MEME‐ChIP. Motifs that had an E‐value ≤ 0.05 were considered significant. Only those TFs that showed at least a two‐fold expression difference between FP4 and SP4 were kept. (**D**) A STRING network of interactions among 46 common target genes regulated by ERF141, MADS7, MADS50 and MYB.
**Figure S7.** Analysis of *Osgata10* overexpression lines and target genes of GATA10
(**A**) Two independent targets within *Osgata10* for construction of *Osgata10* knockout transgenic plants. (**B)** The relative expression levels of *Osgata10, Ub*_*L40*_ *1, Ub*_*L40*_ *2 and Ub*_*L40*_ *4* at the permissive temperature were determined by quantitative RT‐PCR in WXS(F) (as control) and *Osgata10*‐OE transgenic plants. Bar = means ± *SD* from three biological repeats. (**C**) Gene ontology (GO) terms for the target genes regulated by GATA10.
**Figure S8.** Expression profiles of differentially expressed genes (DEGs) encoding kinases during fertility conversion Numbers of DEGs and enrichment of a given kinase gene family are shown. Gene members presenting different expression patterns (clusters C1–C6; see Figure 3) or members of a dedicated gene family are indicated. Enrichment of the gene family was determined by calculating the *P*‐value using Fisher's exact test.
**Figure S9.** KEGG pathways of differentially expressed genes (DEGs)
(**A**) The KEGG pathways of DEGs between SP3 and FP3. (**B**) The KEGG pathways of DEGs between SP4 and FP4.

**Table S1. Comparison of differential Tn5 transposase hypersensitive sites (dTHSs) between WXS(F) and WXS(S) by MAnorm**

**Table S2. The related genes from differential Tn5 transposase hypersensitive sites (dTHSs) by peak annotation in WXS(F) versus WXS(S)**

**Table S3. Differentially expressed genes (DEGs) based on RNA‐seq**

**Table S4. Analysis of the K‐means cluster**

**Table S5. Gene Ontology (GO) analysis of six clusters**

**Table S6. MEME‐ChIP results for WXS(F)‐enriched and WXS(S)‐enriched Tn5 hypersensitive site (THS) sequences**

**Table S7. Motif binding transcription factor expression changes analyzed by DESeq based on our RNA‐seq data**

**Table S8. Coordinates of predicted binding sites in FP3 and SP3 and likely regulated target genes**

**Table S9. Coordinates of predicted binding sites in FP4 and SP4 and likely regulated target genes**

**Table S10. All primers used for this study**
