# Supplementary material for: The transcription factor GATA10 regulates fertility conversion of a two‐line hybrid tms5 mutant rice via the modulation of Ub L40 expression
Source: J Integr Plant Biol. 2019 Nov 6;62(7):1034–56. doi: 10.1111/jipb.12871 (PMC7383616; doi:10.1111/jipb.12871)
Supplement: Supplementary file 1 — Figure S1. ATAC‐seq profiling of nucleus quality, read alignments, fragment size distribution and correlation with published DNaseI hypersensitive sites (A) Fluorescence microscope image of nuclei stained with the DNA‐binding dye DAPI (blue). Scale bar, 100 μm. (B) Mapped reads are all the reads that aligned to the Oryza sativa MSU7 reference genome. Nuclear Mapped Reads exclude the portion of reads that aligned to the mitochondrial or chloroplast genomes; % Aligned = Nuclear Mapped Reads/Clean Reads. Q20 and Q30 denote mapping quality scores. (C) Fragment sizes of ATAC‐seq reads for all four samples (FP3, FP4, SP3, SP4). The dotted line indicates the trendline. (D) Overlapping peaks of Tn5 transposase hypersensitive sites (THSs; this study) and published DHSs in rice. (E) The percent coverage of accessible chromatin regions occupying the rice genome. Figure S2. Correlation of histone modification with Tn5 transposase hypersensitive sites (THSs) by ATAC‐seq (A) Visualization by Integrative Genomics Viewer (IGV) showing enrichment of ATAC‐seq as well as ChIP‐seq in WXS(F) and WXS(S) at P3 and P4. Gene models are displayed on the bottom track. (B) Scatter plots comparing the enrichment between ATAC‐seq and ChIP‐seq. Pearson correlations of the reads per kilobase per million mapped reads (RPKM) values are shown. (C) The profile of H3K9ac and H3K4me2 marks (indicated by the number of ChIP‐seq reads) among genes with different expression levels based on our RNA‐seq data. The expressed genes were divided into five bins from lowest to highest expression. Figure S3. Quantitative real‐time PCR validation of H3K9ac and H3K4me2 ChIP‐seq Fourteen genes were selected for the test (one with reduced, two with unchanged, and four with increased H3K9ac; five with reduced, one with unchanged, and one with increased H3K4me2 in ChIP‐seq, as shown on the left). Levels relative to the input are shown. The DNA level was calculated using the 2−ΔΔCt method. Bar = means ± SD from three biolo [file JIPB-62-1034-s001.pdf]

A

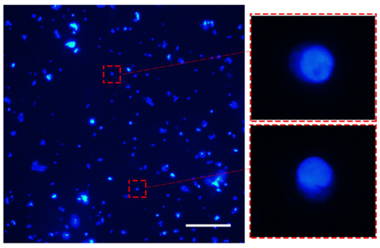

B

Sample	Raw Reads	Clean Reads	Nuclear Mapped Reads (% Aligned)	Q20(%)	Q30(%)
WXS(F)-P3-1	188,691,494	187,009,740	48,222,936(25.79)	99.34	97.60
WXS(F)-P3-2	195,422,392	193,877,024	47,161,734(24.33)	99.37	97.68
WXS(S)-P3-1	181,310,388	179,878,608	42,416,898(23.58)	99.36	97.68
WXS(S)-P3-2	199,027,528	197,394,108	45,843,738(23.22)	99.35	97.65
WXS(F)-P4-1	191,313,180	189,905,502	49,995,394(26.33)	99.48	97.99
WXS(F)-P4-2	214,444,146	212,872,352	55,678,806(26.16)	99.46	97.93
WXS(S)-P4-1	174,606,360	172,798,618	49,146,328(28.44)	99.39	97.79
WXS(S)-P4-2	199,877,888	198,520,566	56,215,486(28.32)	99.48	98.01

C

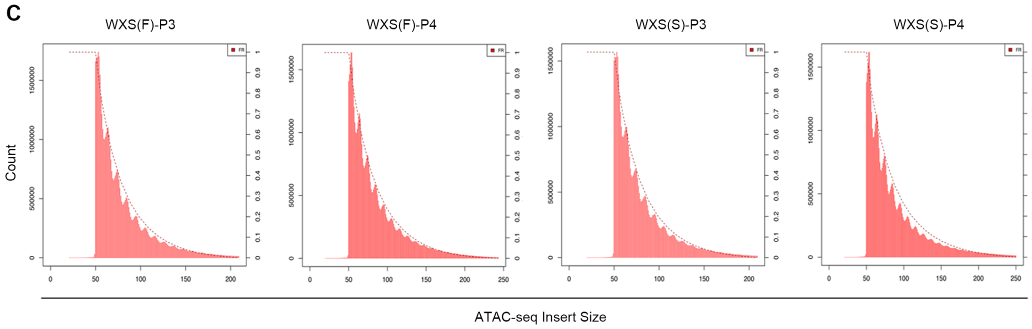

D

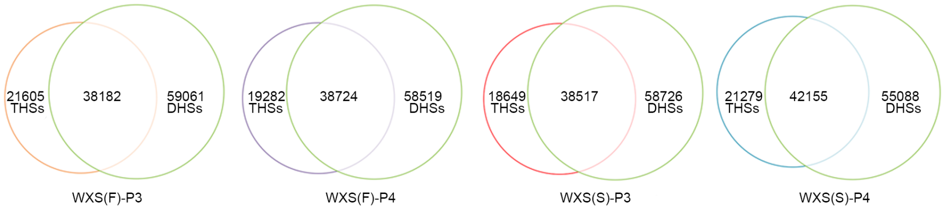

E

Sample	Peak Number	All Peak Length	Genome Size	Open Region Rate (%)
WXS(F)-P3-1	59787	39291700	374305986	10.50
WXS(F)-P3-2	63796	39715352	374305986	10.61
WXS(F)-P4-1	58006	35956601	374305986	9.61
WXS(F)-P4-2	62486	36588030	374305986	9.77
WXS(S)-P3-1	56084	32675977	374305986	8.73
WXS(S)-P3-2	57196	31301884	374305986	8.36
WXS(S)-P4-1	58345	33437130	374305986	8.93
WXS(S)-P4-2	63434	37632945	374305986	10.05

Figure S1. ATAC-seq profiling of nucleus quality, read alignments, fragment size distribution and correlation with published DNaseI hypersensitive sites

(A) Fluorescence microscope image of nuclei stained with the DNA-binding dye DAPI (blue). Scale bar, 100 μm. **(B)** Mapped reads are all the reads that aligned to the *Oryza sativa* MSU7 reference genome. Nuclear Mapped Reads exclude the portion of reads that aligned to the mitochondrial or chloroplast genomes; % Aligned = Nuclear Mapped Reads/Clean Reads. Q20 and Q30 denote mapping quality scores. **(C)** Fragment sizes of ATAC-seq reads for all four samples (FP3, FP4, SP3, SP4). The dotted line indicates the trendline. **(D)** Overlapping peaks of Tn5 transposase hypersensitive sites (THSs; this study) and published DHSs in rice. (**E**) The percent coverage of accessible chromatin regions occupying the rice genome.

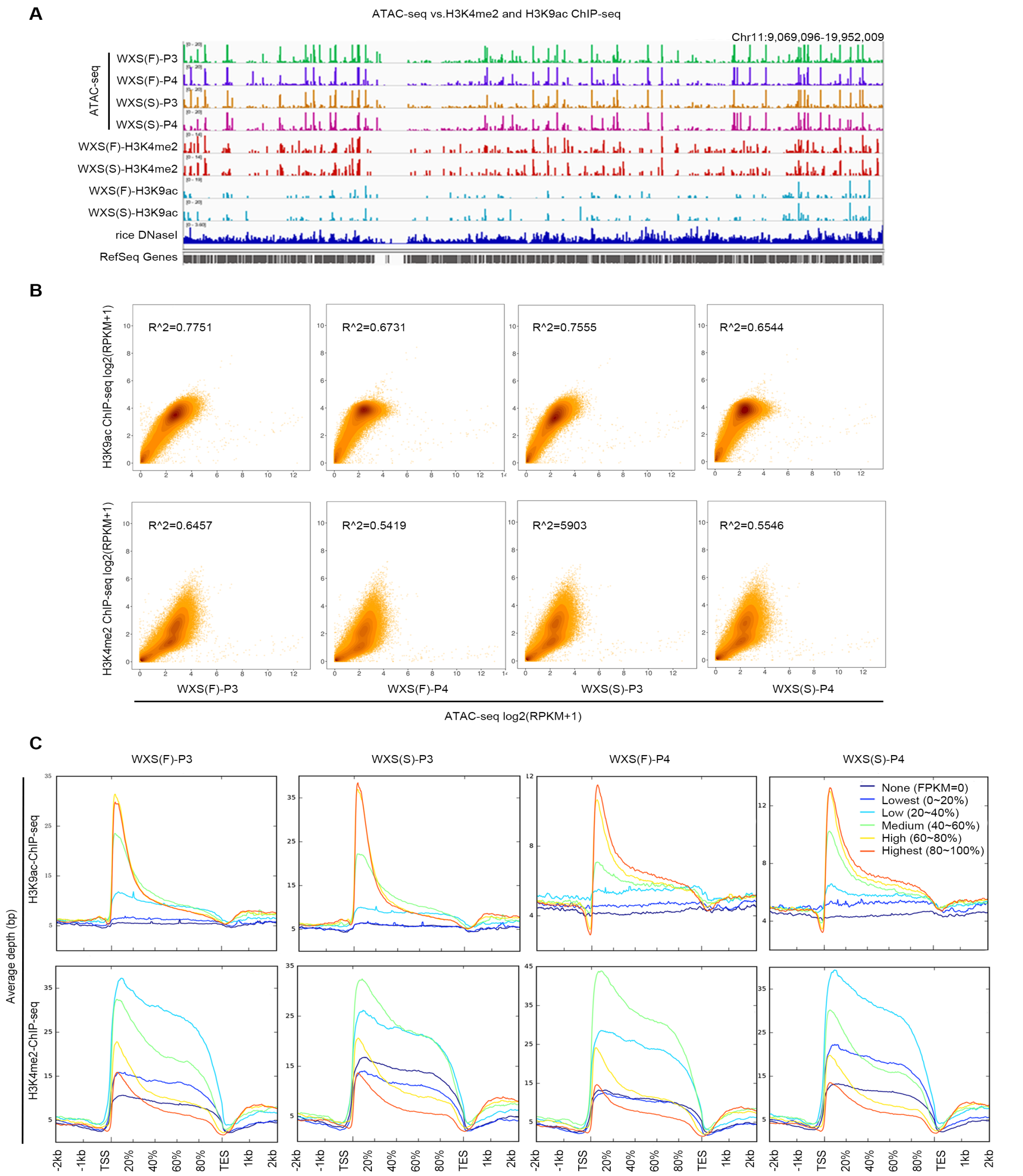

Figure S2. Correlation of histone modification with Tn5 transposase hypersensitive sites (THSs) by ATAC-seq

(A) Visualization by Integrative Genomics Viewer (IGV) showing enrichment of ATAC-seq as well as ChIP-seq in WXS(F) and WXS(S) at P3 and P4. Gene models are displayed on the bottom track. **(B)** Scatter plots comparing the enrichment between ATAC-seq and ChIP-seq. Pearson correlations of the reads per kilobase per million mapped reads (RPKM) values are shown. **(C)** The profile of H3K9ac and H3K4me2 marks (indicated by the number of ChIP-seq reads) among genes with different expression levels based on our RNA-seq data. The expressed genes were divided into five bins from lowest to highest expression.

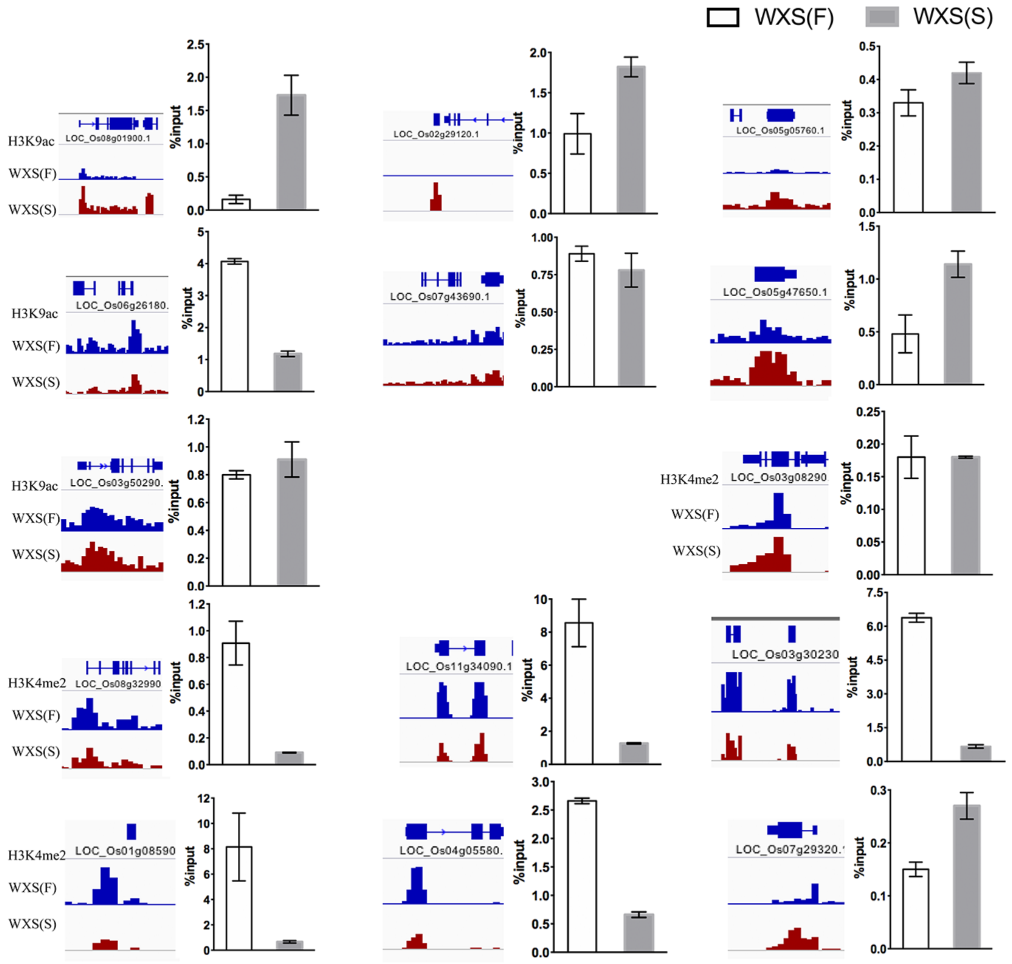

Figure S3. Quantitative real-time PCR validation of H3K9ac and H3K4me2 ChIP-seq

Fourteen genes were selected for the test (1 with reduced, 2 with unchanged, and 4 with increased H3K9ac; 5 with reduced, 1 with unchanged, and 1 with increased H3K4me2 in ChIP-seq, as shown on the left). Levels relative to the input are shown. The DNA level was calculated using the $2^{-\Delta\Delta Ct}$ method. Bar=means +/- SD from three biological repeats.

A

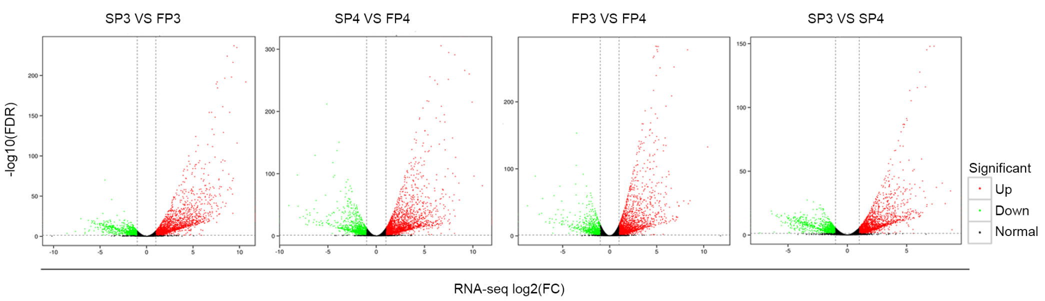

B

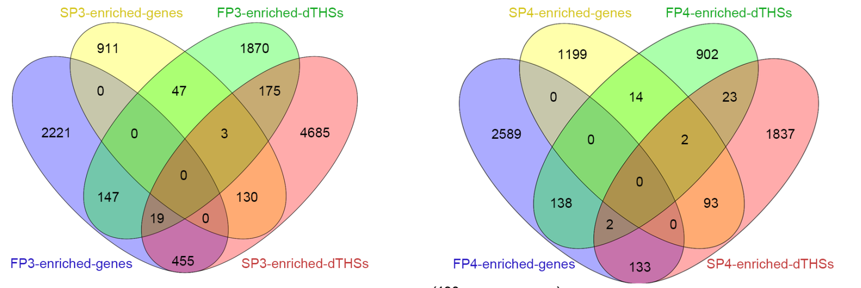

(147 common genes)

Annotation	Number of genes	p-value
transcription regulator activity	20	0.00044
transcription factor activity	20	0.00044
DNA binding	21	0.0089

(455 common genes)

Annotation	Number of genes	p-value
response to stress	98	4.70E-10
response to stimulus	129	7.70E-10
response to abiotic stimulus	57	8.80E-05

(130 common genes)

Annotation	Number of genes	p-value
DNA metabolic process	11	2.60E-06
nucleus	29	2.30E-05
multicellular organismal development	25	6.70E-05
multicellular organismal process	25	9.40E-05
cell cycle	8	0.00015
DNA binding	23	0.00016

(47 common genes)

Annotation	Number of genes	p-value
DNA metabolic process	6	2.80E-05
nitrogen compound metabolic process	14	0.00049
cellular component organization	8	0.00062
nucleus	12	0.00064
nucleic acid binding	12	0.0015

Figure S4. The accessible chromatin regions with all differentially expressed genes (DEGs) from our RNA-seq data

(A) DEGs based on pairwise comparison. Red dots represent upregulated genes, and green dots represent downregulated genes. **(B)** (Left) Venn diagram of overlap between FP3- or SP3-enriched genes and genes associated with FP3- or SP3-enriched differential Tn5 transposase hypersensitive sites (dTHSs). (Right) Venn diagram of overlap between FP4- or SP4-enriched genes and genes associated with FP4- or SP4-enriched dTHSs (right). Genes were considered “enriched” if they had a 2-fold or higher difference in expression between SP3 versus FP3 or SP4 versus FP4. GO analysis was performed to reveal the biological functions of overlapped genes.

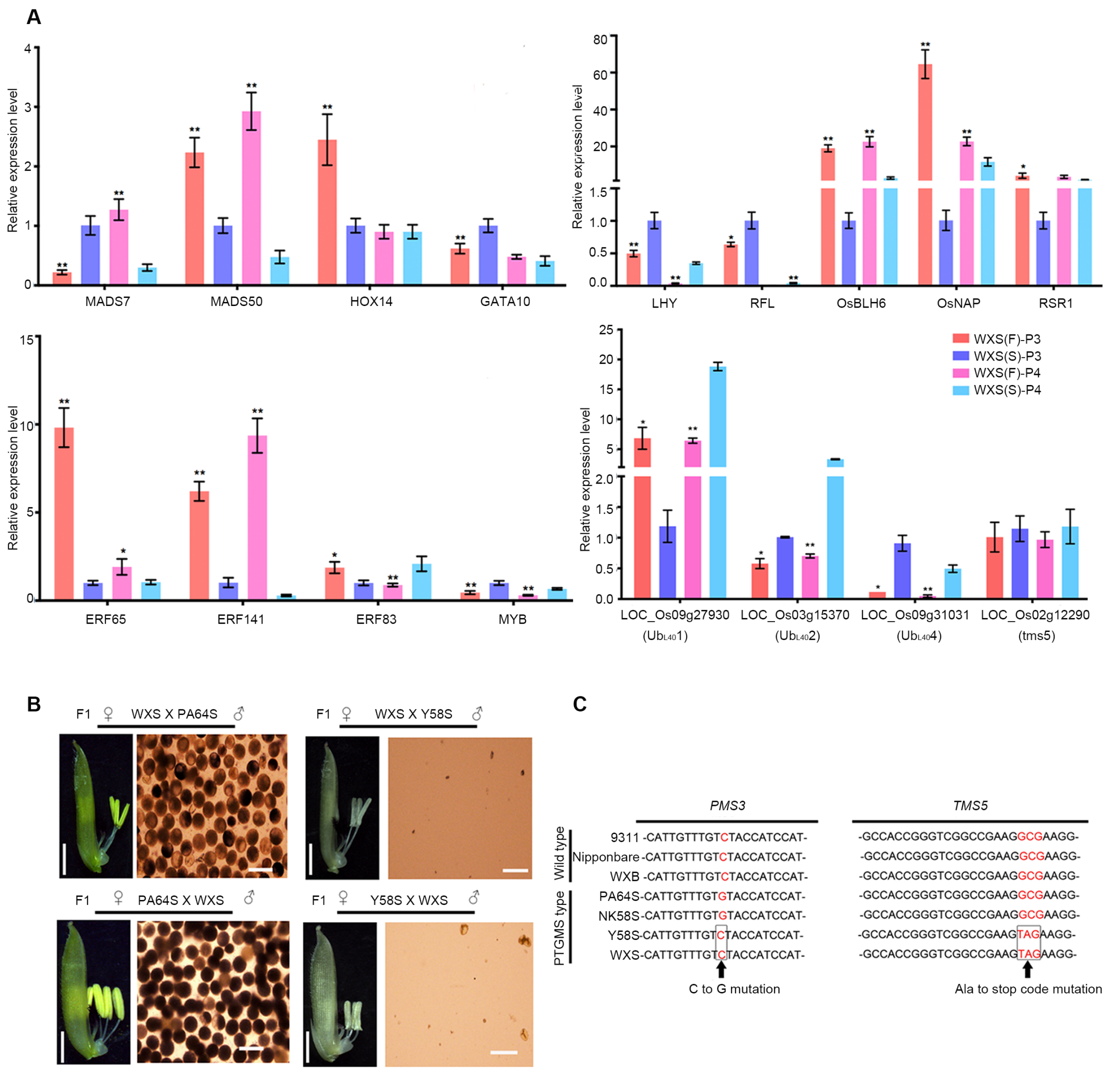

Figure S5. Quantitative real-time PCR verification of RNA-seq results

(**A**) The relative expression levels of 17 genes were determined by quantitative RT-PCR. Significant differences between the samples are indicated by ** (P-value <0.01) and * (P-value <0.05). Bar=means +/- SD from three biological repeats. (**B**) WXS testcross with PA64S and Y58S. The mature anthers of F_1 plants were collected and stained with 1% potassium iodide solution (I_2-KI). Scale bar: 2 mm (left); 20 μm (right). (**C**) The results of SNP site sequencing. Three conventional rice varieties (93-11, Nipponbare and Wuxiang B) and three two-line male sterile lines (PA64S, NK58S and Y58S) as controls were used to perform PCR amplification of the flanking regions of the SNPs. Then, the PCR products were cloned and sequenced.

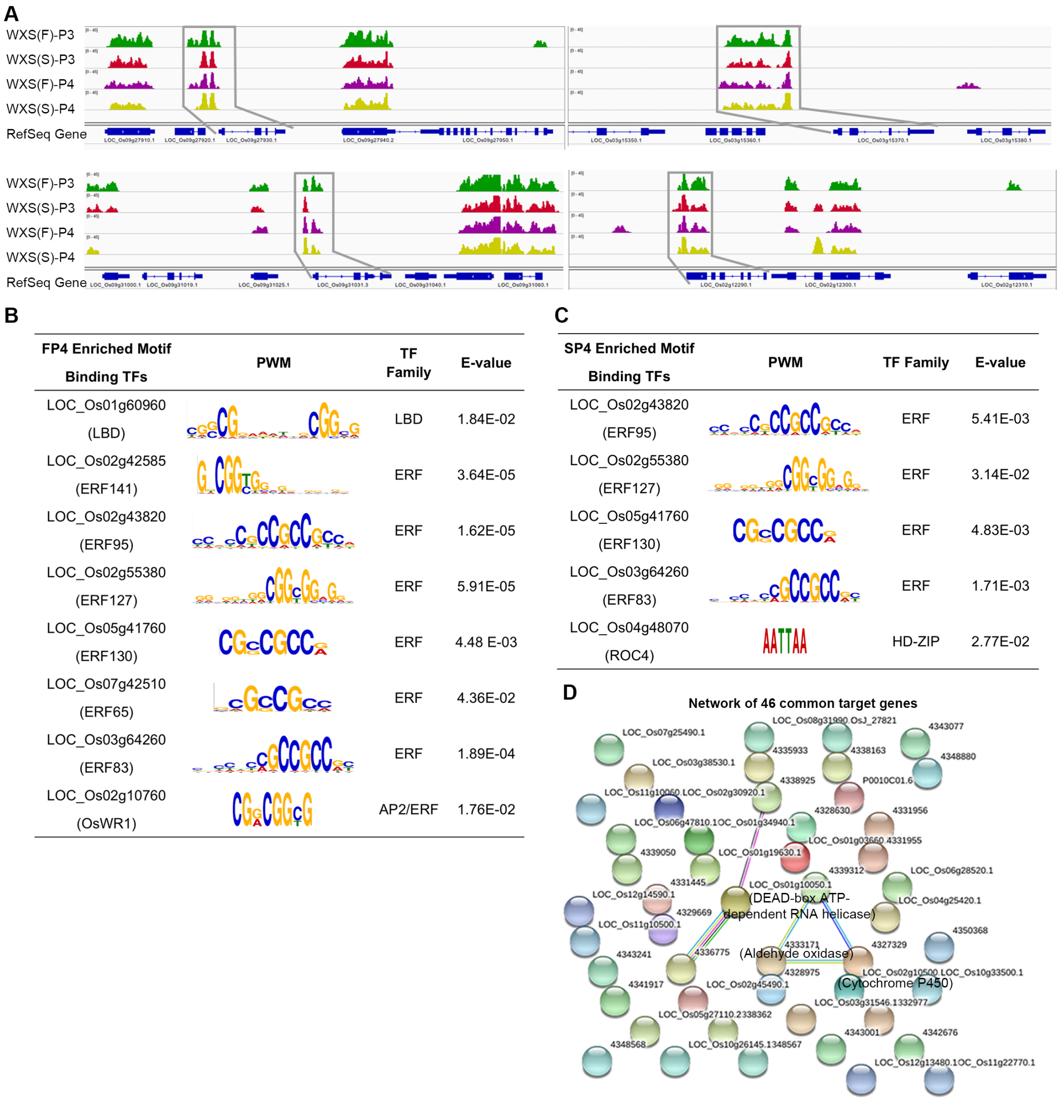

FP4 Enriched Motif Binding TFs	PWM	TF Family	E-value
LOC_Os01g60960 (LBD)		LBD	1.84E-02
LOC_Os02g42585 (ERF141)		ERF	3.64E-05
LOC_Os02g43820 (ERF95)		ERF	1.62E-05
LOC_Os02g55380 (ERF127)		ERF	5.91E-05
LOC_Os05g41760 (ERF130)		ERF	4.48 E-03
LOC_Os07g42510 (ERF65)		ERF	4.36E-02
LOC_Os03g64260 (ERF83)		ERF	1.89E-04
LOC_Os02g10760 (OsWR1)		AP2/ERF	1.76E-02

SP4 Enriched Motif Binding TFs	PWM	TF Family	E-value
LOC_Os02g43820 (ERF95)		ERF	5.41E-03
LOC_Os02g55380 (ERF127)		ERF	3.14E-02
LOC_Os05g41760 (ERF130)		ERF	4.83E-03
LOC_Os03g64260 (ERF83)		ERF	1.71E-03
LOC_Os04g48070 (ROC4)		HD-ZIP	2.77E-02

Figure S6. Predicted sequence motifs and target genes for WXS(F)- and WXS(S)-enriched transcription factors (TFs) in the uninucleate period (P4)

(**A**) Accessible chromatin profiling of *Ub*$_{L40}$*1, Ub*$_{L40}$*2, Ub*$_{L40}$*4* and *tms5* in FP3, SP3, FP4 and SP4. (**B**) FP4-enriched Tn5 transposase hypersensitive site (THS) sequences and (**C**) SP4-enriched THS sequences were analyzed using MEME-ChIP. Motifs that had an E-value ≤ 0.05 were considered significant. Only those TFs that showed at least a 2-fold expression difference between FP4 and SP4 were kept. (**D**) A STRING network of interactions among 46 common target genes regulated by ERF141, MADS7, MADS50 and MYB.

A

Target 1 GTGGATCCCGACGAGTGCAACGG

Target 2 GCGCCAGAGGTTGATTCGGTTGG

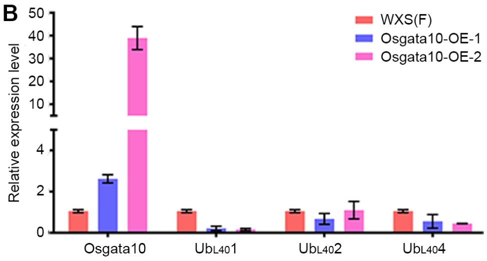

C

GO term	Description	Numbers	p-value	FDR
GO:0043176	amine binding	5	1.4E-04	4.2E-03
GO:0016597	amino acid binding	5	1.4E-04	4.2E-03
GO:0031406	carboxylic acid binding	5	8.1E-04	1.3E-02
GO:0016491	oxidoreductase activity	30	8.6E-04	1.3E-02

Figure S7. Analysis of *Osgata10* overexpression lines and target genes of GATA10

(**A**) Two independent targets within *Osgata10* for construction of *Osgata10* knockout transgenic plants. (**B)** The relative expression levels of *Osgata10, Ub$_{L40}$1, Ub$_{L40}$2 and Ub$_{L40}$4* at the permissive temperature were determined by quantitative RT-PCR in WXS(F) (as control) and *Osgata10*-OE transgenic plants. Bar=means +/- SD from three biological repeats. (**C**) Gene Ontology (GO) terms for the target genes regulated by GATA10.

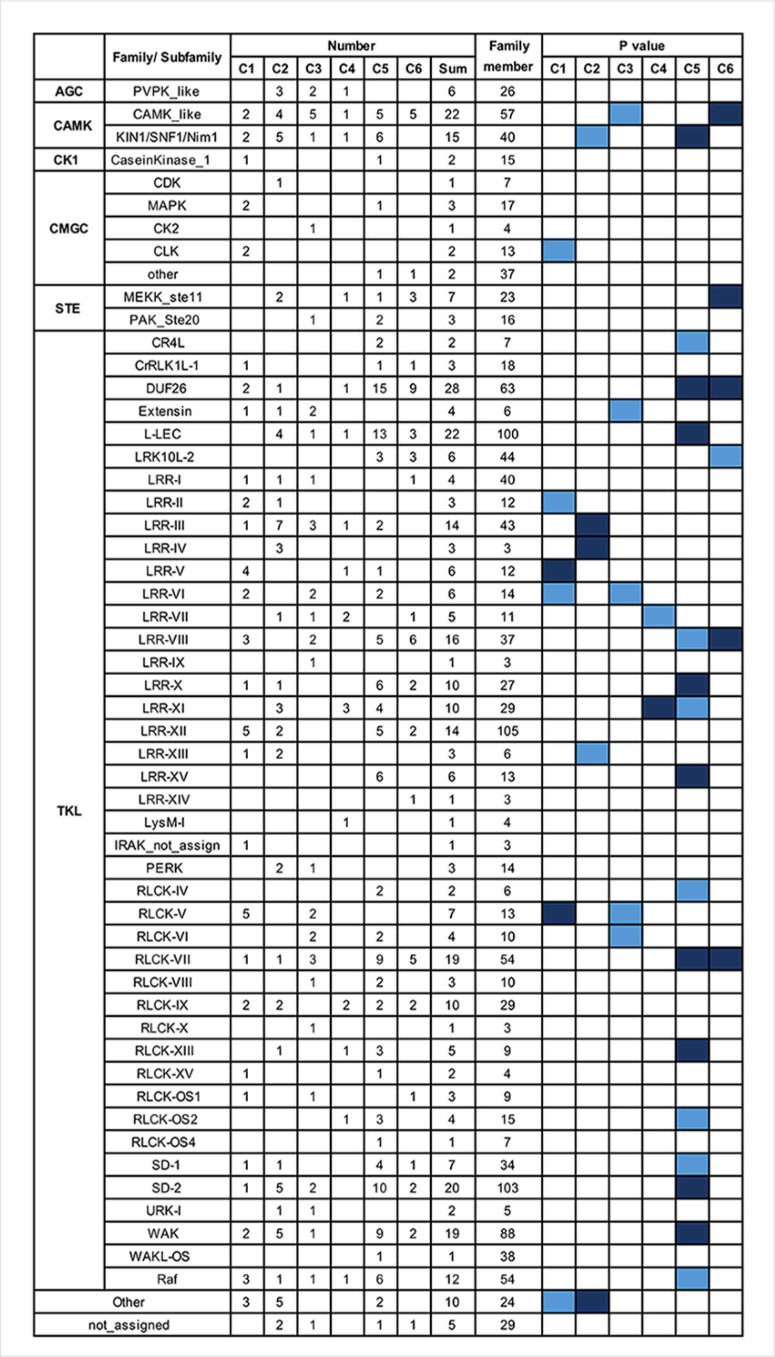

	Family/ Subfamily	Number							Family member	P value					
		C1	C2	C3	C4	C5	C6	Sum		C1	C2	C3	C4	C5	C6
AGC	PVPK_like		3	2	1			6	26						
CAMK	CAMK_like	2	4	5	1	5	5	22	57						
	KIN1/SNF1/Nim1	2	5	1	1	6		15	40						
CK1	CaseinKinase_1	1				1		2	15						
CMGC	CDK		1					1	7						
	MAPK	2				1		3	17						
	CK2			1				1	4						
	CLK	2						2	13						
	other					1	1	2	37						
STE	MEKK_ste11		2		1	1	3	7	23						
	PAK_Ste20			1		2		3	16						
TKL	CR4L					2		2	7						
	CrRLK1L-1	1				1	1	3	18						
	DUF26	2	1		1	15	9	28	63						
	Extensin	1	1	2				4	6						
	L-LEC		4	1	1	13	3	22	100						
	LRK10L-2					3	3	6	44						
	LRR-I	1	1	1			1	4	40						
	LRR-II	2	1					3	12						
	LRR-III	1	7	3	1	2		14	43						
	LRR-IV		3					3	3						
	LRR-V	4			1	1		6	12						
	LRR-VI	2		2		2		6	14						
	LRR-VII		1	1	2		1	5	11						
	LRR-VIII	3		2		5	6	16	37						
	LRR-IX			1				1	3						
	LRR-X	1	1			6	2	10	27						
	LRR-XI		3		3	4		10	29						
	LRR-XII	5	2			5	2	14	105						
	LRR-XIII	1	2					3	6						
	LRR-XV					6		6	13						
	LRR-XIV						1	1	3						
	LysM-I				1			1	4						
	IRAK_not_assign	1						1	3						
	PERK		2	1				3	14						
	RLCK-IV					2		2	6						
	RLCK-V	5		2				7	13						
	RLCK-VI			2		2		4	10						
	RLCK-VII	1	1	3		9	5	19	54						
	RLCK-VIII			1		2		3	10						
	RLCK-IX	2	2		2	2	2	10	29						
	RLCK-X			1				1	3						
	RLCK-XIII		1		1	3		5	9						
	RLCK-XV	1				1		2	4						
	RLCK-OS1	1		1			1	3	9						
	RLCK-OS2				1	3		4	15						
	RLCK-OS4					1		1	7						
	SD-1	1	1			4	1	7	34						
	SD-2	1	5	2		10	2	20	103						
	URK-I		1	1				2	5						
	WAK	2	5	1		9	2	19	88						
	WAKL-OS					1		1	38						
	Raf	3	1	1	1	6		12	54						
Other		3	5			2		10	24						
not_assigned			2	1		1	1	5	29						

Figure S8. Expression profiles of differentially expressed genes (DEGs) encoding kinases during fertility conversion

Numbers of DEGs and enrichment of a given kinase gene family are shown. Gene members presenting different expression patterns (clusters C1–C6; see Figure 3) or members of a dedicated gene family are indicated. Enrichment of the gene family was determined by calculating the P-value using Fisher's exact test.

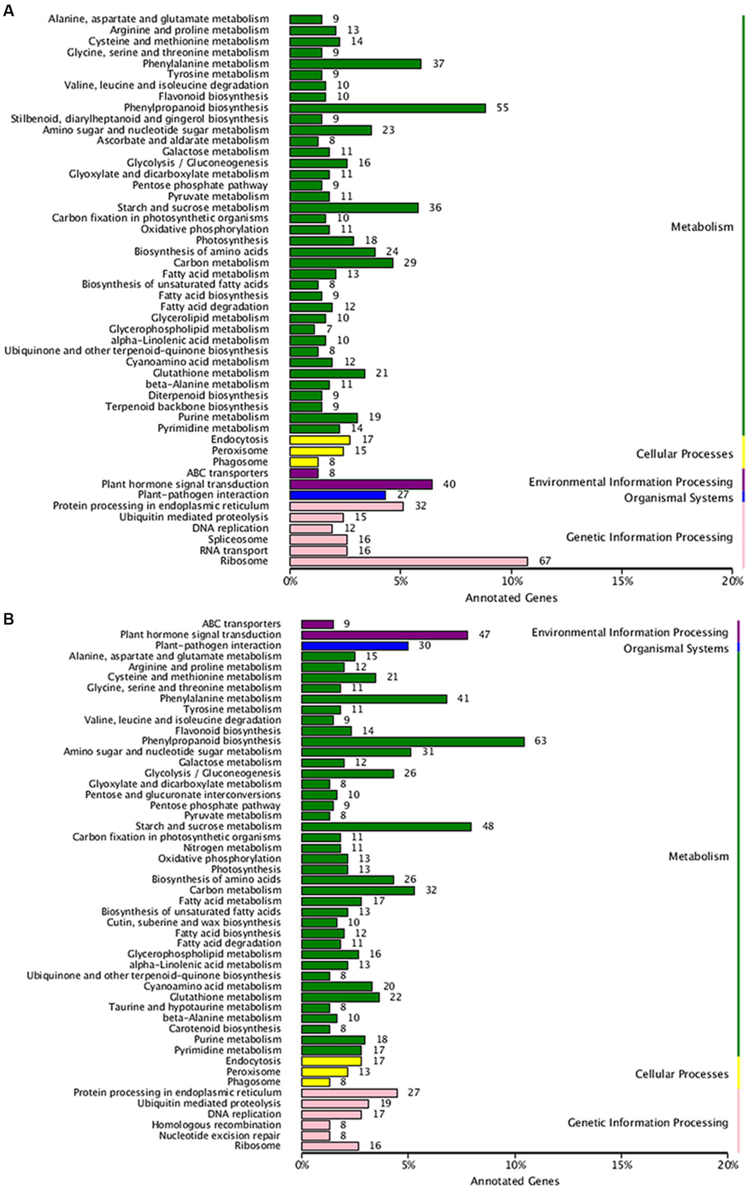

Figure S9. KEGG pathways of differentially expressed genes (DEGs)

(**A**) The KEGG pathways of DEGs between SP3 and FP3. (**B**) The KEGG pathways of DEGs between SP4 and FP4.
